# Structure based discovery of VMP-7 as a promising GlgE targeting lead against *Mycobacterium tuberculosis*

**DOI:** 10.3389/fmicb.2026.1909418

**Published:** 2026-09-07

**Authors:** Pratik Mahajan, Sushama Jadhav, Amitkumar Singh, Anupam Mukherjee, Manali Joshi, Vijay Nema

**Affiliations:** 1 Division of Molecular Biology, ICMR - National Institute of Translational Virology and AIDS Research, Pune, Maharashtra, India; 2 Krishna Institute of Science and Technology, Krishna Vishwa Vidyapeeth (Deemed to be University), Karad,Maharashtra India; 3 Bioinformatics Centre, Savitribai Phule Pune University, Pune, Maharashtra, India; 4 Experimental Animal Facility, ICMR-National Institute for Research in Tuberculosis (NIRT-JALMA, North India Campus), Agra, Uttar Pradesh, India; 5 Academy of Scientific and Innovative Research, Ghaziabad, Uttar Pradesh, India; 6 ICMR-National Institute of Virology, Pune, Maharashtra, India; 7 ICMR-National Institute for Tribal Health Research, Jabalpur, Madhya Pradesh, India

**Keywords:** drug discovery, high throughput virtual screening, Maltosyltransferase (GlgE), *Mycobacterium tuberculosis*, tubercuolosis, VMP-7

## Abstract

Tuberculosis (TB), caused by *Mycobacterium tuberculosis* (*M.tb*), remains a major global health challenge, necessitating the identification of novel drug targets with high specificity and therapeutic potential. Maltosyltransferase (GlgE) is a critical enzyme in the biosynthesis of cytosolic α-glucan, a key component required for maintaining cell wall integrity and intracellular survival of the pathogen. GlgE catalyzes the transfer of maltosyl units from maltose-1-phosphate to elongating α-glucan chains, and its inactivation results in toxic accumulation of metabolic intermediates, ultimately leading to bacterial death. Importantly, the absence of homologous pathways in humans underscores its suitability as a selective anti-tubercular (anti-TB) target. Sequence analysis of 382 clinical isolates demonstrated complete conservation of the *glgE* gene, highlighting its evolutionary stability and essentiality. Subsequently, structure-based virtual screening followed by molecular fingerprint clustering was performed to identify chemically diverse inhibitors. Twelve top ranked compounds (VMP-1 to VMP-12) were further evaluated through *in-vitro* antimycobacterial assays in broth culture and infected THP-1 derived macrophages, along with cytotoxicity assessment. Among these, VMP-7 exhibited significant inhibitory activity against *M.tb* at 12.5 μg/mL, with minimal cytotoxic effects on host cells. Collectively, these findings validate GlgE as a highly conserved target and identify VMP-7 as a promising lead candidate for further preclinical development of novel anti-TB therapeutics.

## 1 Introduction

Tuberculosis (TB), caused by *Mycobacterium tuberculosis* (*M.tb*), remains one of the leading causes of death from infectious diseases worldwide. Despite sustained global control efforts, TB continues to impose a substantial public health burden (Suvvari, 2025). As per the Global Tuberculosis Report 2025, it is estimated that there were 10.7 million TB cases and 1.23 million deaths in 2024 (World Health Organization, 2025). The particular concern is the escalating prevalence of drug resistant TB, which threatens the efficacy of current therapeutic regimens. Globally in 2024, 147,592 MDR/RR-TB cases and 25,140 pre-XDR-TB or XDR-TB cases were detected among those tested for drug resistance (World Health Organization, 2025).

The emergence of drug resistance in *M.tb* is multifactorial, driven by prolonged treatment regimens, poor patient adherence, inadequate healthcare infrastructure, and co-infection with HIV. Standard TB chemotherapy requires a minimum of 6 months, increasing the likelihood of incomplete treatment and subsequent selection of resistant strains (Mahajan et al., 2023). A major limitation is that over time, *M.tb* has evolved sophisticated mechanisms to evade these drugs, including target modification, efflux systems, and metabolic adaptation (Nguyen, 2016). Moreover, the intrinsic ability of *M.tb* to persist in a dormant or latent state further complicates eradication, as most frontline antibiotics target actively replicating bacilli (Jadhav et al., 2024). Collectively, these challenges underscore the urgent need for novel therapeutic strategies capable of overcoming resistance, shortening treatment duration, and targeting both replicating and non-replicating bacterial populations.

Structure based drug discovery has emerged as a powerful strategy for identifying novel inhibitors against identified targets. Central to this approach is molecular docking, a computational technique that predicts the binding orientation and affinity of small molecules within the active site of a target protein (Ferreira et al., 2015). By simulating protein-ligand interactions at the atomic level, docking enables the rapid screening of large chemical libraries and prioritization of candidate compounds based on predicted binding energies and interaction profiles (Wei and McCammon, 2024). High throughput virtual screening (HTVS) workflows typically employ hierarchical docking strategies to balance computational efficiency and accuracy, allowing the identification of high affinity ligands from extensive compound libraries. The integration of post docking analyses, such as binding free energy estimation, further enhances the reliability of hit selection (Ferreira et al., 2015). Compared to traditional experimental screening, HTVS offers significant advantages in terms of cost, speed, and the ability to rationally design and optimize lead compounds (Shaik and Balya, 2025).

In the present study, we focus on Maltosyltransferase (GlgE), a key enzyme in the α-glucan biosynthesis pathway of *M.tb*. The GlgE pathway represents a non-classical metabolic route that converts trehalose into branched α-glucan. GlgE catalyzes the transfer of maltosyl units from maltose-1-phosphate (M1P) to elongating α-glucan chains, thereby playing a central role in maintaining cellular homeostasis and cell wall-associated processes (Bornemann, 2016). GlgE has been genetically validated as an essential enzyme for *M.tb* survival. Notably, its inhibition results in a unique bactericidal mechanism termed “self-poisoning,” wherein the accumulation of the substrate M1P reaches toxic levels within the bacterial cell. This accumulation induces a cascade of metabolic stress responses, ultimately leading to rapid cell death (Kalscheuer et al., 2010). Previous studies have provided important proof-of-concept for targeting GlgE as an anti-TB drug target. Veleti and co-workers reported the first transition-state GlgE inhibitor, 2,5-dideoxy-3-O-α-D-glucopyranosyl-2,5-imino-D-mannitol (DDGIM; compound 9) which is a poly-hydroxypyrolidine-based inhibitor, which inhibited recombinant *M.tb* GlgE with a Ki of 237 ± 27 μM, comparable to the reported M1P Km of approximately 250 μM. The proposed mechanism involved inhibiting glycoside hydrolases activity, with the protonated secondary ammonium group predicted to interact with the catalytic Asp418 residue (Veleti et al., 2014). Subsequent crystallographic studies provided structural evidence for GlgE inhibitor recognition and demonstrated that the non-hydrolysable M1P analog α-maltose-C-phosphonate (MCP) inhibited Mtb GlgE with an IC50 of 237 ± 24 μM, whereas DDGIM showed a comparable IC50 of 237 ± 27 μM. The DDGIM-GlgE structure revealed occupation of the −1 subsite where the reducing end of maltose or the phospho-glucosyl moiety of the M1P substrate binds, an ionic interaction between its secondary ammonium group and catalytic Asp418 at ~2.6 Å, and extensive hydrogen-bonding interactions within the −2 subsite where the non-reducing glucosyl moiety is bound, highlighting this region as an important determinant of inhibitor recognition (Lindenberger et al., 2015). Notably, these pioneering compounds primarily demonstrated biochemical GlgE inhibition, but the reported studies did not establish whole cell anti-TB MIC values for DDGIM or MCP.

Importantly, GlgE satisfies several key criteria for an ideal drug target. It is essential for bacterial viability and lacks homologous counterparts in humans. Additionally, detailed structural and biochemical studies have elucidated the architecture of its active site and catalytic mechanism, providing a strong framework for HTVS led identification (Lindenberger et al., 2015). These features collectively establish GlgE as a highly attractive target for the development of novel anti-TB agents.

In this context, the present study employs an integrated strategy combining computational and experimental approaches to identify and validate novel GlgE inhibitors. First, the evolutionary conservation of the *glgE* gene was assessed across a diverse set of clinical isolates and global *M.tb* genomes to evaluate its stability and suitability as a universal drug target (Mahajan et al., 2026). Subsequently, a structure based HTVS approach was implemented using a large library of natural product derivatives. The crystal structure of GlgE was utilized to define the catalytic binding pocket in the active site, and a hierarchical docking workflow was employed to identify compounds with high binding affinity and favorable interaction profiles (Lindenberger et al., 2015).

To validate the computational findings, top ranked compounds were subjected to *in-vitro* antimycobacterial assays, including determination of minimum inhibitory concentrations (MICs). Furthermore, the intracellular efficacy of selected candidates was evaluated using THP-1 derived macrophage infection models, providing insight into their ability to inhibit *M.tb* within host cells. Cytotoxicity assessments were also performed to ensure selective activity against the pathogen without detrimental effects on host cells. Collectively, this integrated computational and experimental approach identified a promising GlgE inhibitor with significant antimycobacterial activity and favorable host-cell safety, highlighting its potential for further lead optimization and preclinical development against drug-resistant TB.

## 2 Materials and methods

### 2.1 Bacterial clinical isolates

A total of 72 *M.tb* isolates were obtained from three geographically distinct regions of India. These comprised YM 1 to YM 18 and BJ 1 to BJ 12 from Pune (western India), CH 1 to CH 28 from Chennai (southern India), and B 1 to B 14 from Varanasi (northern India). In addition to these clinical isolates, publicly available genomic datasets were retrieved from the BV-BRC database (Shukla et al., 2026). This resource includes 310 fully sequenced *M.tb* genomes derived from human clinical specimens, each associated with high-quality sequencing datasets, defined geographic origin, and corresponding phenotypic drug susceptibility testing (DST) profiles. All experiments involving *M.tb* isolates were conducted in a certified BSL-3 facility following institutional biosafety guidelines.

### 2.2 *glgE* gene amplification and sequencing

Primers targeting the *glgE* gene were designed using the Primer3web platform (Untergasser et al., 2012) to ensure optimal specificity and amplification efficiency. Genomic DNA was isolated from bacterial isolates using a modified cetyltrimethylammonium bromide (CTAB) extraction protocol (De Almeida et al., 2013), yielding high-quality DNA. Briefly, bacterial cells were lysed in a Lysozyme and CTAB containing buffer supplemented with Proteinase K, SDS, and NaCl, followed by protein removal using chloroform:isoamyl alcohol (24:1) extraction. The aqueous phase containing DNA was subsequently recovered and precipitated with cold isopropanol, washed to remove residual salts and contaminants, and finally resuspended in TE buffer to obtain high-quality genomic DNA. A 2106 bp fragment of the *glgE* gene was amplified by polymerase chain reaction (PCR) using the forward primer GlgE-66F (5′-GCGGGTGTGATCGGATACTA-3′) and the reverse primer GlgE+100R (5′-GTGCGGGTTGTGATGTGTAC-3′). PCR reactions were performed with KAPA HiFi DNA Polymerase (#KK2102) under optimized amplification conditions, with an annealing temperature of 65°C to ensure high specificity and minimal amplification errors.

The resulting amplicons were purified and subjected to bidirectional sequencing using the BigDye Terminator v3.1 Cycle Sequencing Kit (#4337455) according to the manufacturer's guidelines. Capillary electrophoresis was carried out on an ABI 3730xl DNA Analyzer to generate high-resolution sequence data. To achieve complete and accurate coverage of the target region, additional internal sequencing primers were employed, including GlgE316F (5′-GTTTTCCACGGCCAGTTCAC-3′), GlgE1271R (5′-GGTTTGGTGTGGGGATTGTC-3′) and GlgE1675R (5′-CGTACTTCTCCGAGTCCAGG-3′). Sequencing reactions using these primers were performed at an annealing temperature of 54°C, generating overlapping reads across the entire gene.

### 2.3 Sequence analysis

Sequencing chromatograms were processed and assembled using Applied Biosystem's SeqScape software v2.7 for contig generation, variant identification, and sequence interpretation. Ambiguous base calls were resolved and were manually curated through visual inspection of electropherograms. The curated sequences were subsequently aligned against the reference *glgE* gene (Rv1327c) from *M.tb* H37Rv available in the Mycobrowser (Kapopoulou et al., 2011) to generate the full-length consensus *glgE* sequence.

### 2.4 SNP analysis of BV-BRC database derived genomic data

Raw sequencing data for 310 *M.tb* isolates were retrieved in FASTQ format from the BV-BRC database. Initial quality assessment of the reads was performed using FastQC implemented within the Galaxy environment. High-quality reads were subsequently processed using the Bowtie2 package (Langmead and Salzberg, 2012), where indexing was carried out against the reference *glgE* gene (Rv1327c) from the H37Rv strain. The filtered reads were aligned to the reference sequence, followed by variant detection and single nucleotide polymorphism (SNP) analysis using SAMtools and BCFtools (Danecek et al., 2021).

### 2.5 Structure based high throughput virtual screening of inhibitory compounds

A structure guided HTVS strategy was implemented to identify small molecules targeting the active sites of GlgE. A library of ~163,000 natural product derived compounds was sourced from the TargetMol^@^ Natural Product Derivative Library (Cat No L6030) for screening. Ligand preparation was carried out using Schrödinger (LigPrep module), including generation of three-dimensional structures, assignment of bond orders, addition of hydrogens, stereochemical correction, and removal of salts. Ionization and tautomeric states were generated at physiological pH, followed by energy minimization using the OPLS force field (Harder et al., 2016). The crystal structure of GlgE (PDB ID: 4U33; Lindenberger et al., 2015) was obtained from the Protein Data Bank and processed using the Protein Preparation Wizard in Schrödinger. Protein preparation involved removal of non-essential water molecules and heteroatoms, addition of missing hydrogens, optimization of hydrogen-bonding networks, assignment of appropriate protonation states, and restrained energy minimization. A docking grid was defined around the catalytic residues of the target protein, with a dimension of 10 Å^3^ centered on the active-site pocket. Molecular docking was performed using the Glide module in a hierarchical workflow comprising HTVS, Standard Precision (SP), and Extra Precision (XP) modes. Compounds were sequentially filtered, with top-ranked candidates from each stage advanced to the next level to improve accuracy in binding pose prediction and scoring. The binding affinities of the top XP-ranked complexes were further evaluated using the Molecular Mechanics Generalized Born Surface Area (MM-GBSA) approach to estimate relative binding free energies. MM-GBSA combines molecular mechanics energy terms, including van der Waals and electrostatic interactions, with polar solvation energy calculated using the Generalized Born model and non-polar solvation contributions estimated from the solvent-accessible surface area. The resulting binding free-energy estimate (ΔG-bind) provides a quantitative measure of ligand–protein binding favorability, with more negative values generally indicating stronger predicted binding (Dasmahapatra et al., 2022). Finally, selected hits were clustered based on molecular fingerprint similarity to identify representative scaffolds and eliminate redundancy, yielding a refined set of chemically diverse candidate inhibitors. Based on docking scores, MM-GBSA binding energies, and scaffold diversity, twelve compounds were shortlisted for experimental validation.

### 2.6 *In vitro* evaluation of antimycobacterial activity and determination of minimum inhibitory concentration

The antimycobacterial activity of compounds VMP-1 to VMP-12 was evaluated using a resazurin-based viability assay (Invitrogen Alamar Blue Assay #DAL1025), which quantifies metabolic activity through the reduction of resazurin to resorufin. Log-phase cultures of *M.tb* H37Rv, corresponding to an OD_600_ of 0.6, were standardized to 1 × 10^5^ CFU/mL in Middlebrook 7H9 medium supplemented with 10% ADC. Aliquots (100 μL) of the bacterial suspension were dispensed into sterile 96-well microtiter plates, followed by the addition of test compounds to achieve final concentrations of 250, 125, and 50 μg/mL in a total reaction volume of 200 μL per well. Isoniazid (INH) was included as a positive control, while untreated bacterial cultures served as growth controls, and 1% (v/v) DMSO was maintained as the solvent control.

The plates were incubated statically at 37°C for 10 days, after which 20 μL of Alamar Blue reagent was added to each well and incubation was continued for an additional 24 h. A transition in color from blue to pink was interpreted as active bacterial metabolism, whereas retention of the blue color indicated inhibition of bacterial activity. Quantitative assessment of bacterial viability was performed by measuring absorbance at 550 nm and 610 nm using a microplate reader.

Compounds demonstrating significant inhibitory activity in the primary screening were further subjected to MIC determination using a two-fold serial dilution method. Concentrations ranging from 50 to 1.62 μg/mL were tested under identical experimental conditions. The MIC was defined as the lowest concentration at which no detectable color transition from blue to pink was observed, indicating complete inhibition of bacterial growth.

### 2.7 Cytotoxicity assays

The cytotoxic effects of test compounds on host cells were evaluated using the 3-(4,5-dimethylthiazol-2-yl)-2,5-diphenyltetrazolium bromide (MTT) colorimetric assay, which quantifies cellular metabolic activity through the reduction of the tetrazolium salt MTT into insoluble formazan crystals by mitochondrial enzymes in viable cells (Jain et al., 2018). Differentiated THP-1 derived macrophages were seeded in 96-well plates at a density of 1 × 10^4^ cells per well and incubated overnight at 37°C in a humidified atmosphere containing 5% CO_2_ to allow adherence.

Cells were exposed to test compounds at concentrations corresponding to intracellular efficacy experiments. Untreated cells were maintained as viability controls (100% viability), while DMSO-treated wells served as vehicle controls. Each condition was evaluated in triplicate. Following treatment, plates were incubated for 24, 48, and 72 h under standard culture conditions.

At each time point, 20 μL of MTT solution (5 mg/mL in PBS) was added to each well to achieve a final concentration of 0.5 mg/mL, followed by incubation for 4 h in the dark to allow formazan crystal formation. The supernatant was carefully removed, and the crystals were solubilized using 100 μL of DMSO per well. Plates were gently agitated and incubated at room temperature for 30 min to ensure complete dissolution.

Absorbance was recorded at 550 nm with a reference wavelength of 630 nm using a microplate reader, and corrected absorbance values were calculated as OD_550_ – OD_630_. Percentage cell viability was determined using the equation:

$$\%\text{cell Viability =}\frac{\left({\text{OD}}_{\text{sample}}{\text{-OD}}_{\text{blank}}\right)}{\left({\text{OD}}_{\text{control}}{\text{-OD}}_{\text{blank}}\right)}\times \text{100}$$

where ODsample corresponds to treated cells, ODcontrol to untreated cells, and ODblank to medium only wells. The resulting viability profiles were used to assess compound-associated cytotoxicity and determine their suitability for subsequent intracellular efficacy studies. All experiments were performed using at least three independent biological replicates, each with technical triplicates to ensure reproducibility.

### 2.8 Intracellular inhibition assays in THP-1 derived macrophages

Human THP-1 monocytes were differentiated into macrophages using stimulation of 50 ng/ml Phorbol 12-myristate 13-acetate for 24 h and subsequently infected with *M.tb* H37Rv and a multidrug-resistant (MDR) strain (exhibited resistance to isoniazid and rifampicin as determined by MGIT 960 method). At a multiplicity of infection (MOI) of 10:1, using an initial bacterial load of ~1 × 10^5^ CFU/mL Infection was carried out for 4 h at 37°C in a 5% CO_2_ atmosphere to allow efficient bacterial internalization (Baxter et al., 2020). Following infection, extracellular bacteria were removed by repeated washing with pre-warmed PBS, and cells were maintained in RPMI-1640 medium supplemented with 10% fetal bovine serum (FBS).

To evaluate the intracellular antimycobacterial activity of VMP-7, *M.tb* infected THP-1 derived macrophages were treated with VMP-7 at two different concentrations, 25 and 50 μg/mL, based on MIC derived dosing. INH was used as a positive control, while infected but untreated cells served as negative controls. Cultures were incubated at 37°C with 5% CO_2_, and intracellular bacterial survival was evaluated at 48 and 72 h post-treatment.

At each time point, macrophages were lysed using 0.05% SDS in PBS to release intracellular bacteria. The lysates were serially diluted and plated onto Middlebrook 7H10 agar plates, followed by incubation at 37°C. Colony-forming units (CFUs) were enumerated after 7 days of incubation periods. Intracellular bacterial burden was quantified based on CFU counts, enabling assessment of the antimicrobial efficacy of the test compound.

To quantify drug interactions, the Synergy Index (SI) was calculated using the Loewe additivity model based on normalized bacterial survival. SI < 1 indicated synergy, SI = 1 additive interaction, and SI > 1 antagonism. This analysis was used to characterize the combinatorial effects of VMP-7 with standard anti-TB drugs at different treatment durations (Kashif et al., 2017). All experiments were performed in triplicate to ensure reproducibility and statistical reliability.

## 3 Result

### 3.1 *glgE* gene reveals complete sequence conservation

Sequence conservation analysis performed across the combined dataset of 382 *M.tb* isolates, comprising 72 clinical isolates from India and 310 globally derived genomes from the BV-BRC database, demonstrated complete conservation of the *glgE* gene. No nucleotide variations were detected across the full-length coding sequence, with an absence of both synonymous and non-synonymous substitutions in all analyzed isolates. This remarkable lack of genetic variability indicates that the *glgE* locus is highly conserved across geographically and genetically diverse strains of *M.tb*. The absence of synonymous and non-synonymous substitutions suggests that *gIgE* is under strong purifying selection. Such complete conservation across a large and diverse dataset is indicative of strong purifying selection, reflecting the critical functional importance of this gene in bacterial physiology. Unlike many other metabolic genes that exhibit some degree of polymorphism, the invariant nature of *glgE* underscores its indispensable role in maintaining cellular homeostasis. From a functional perspective, GlgE is a key enzyme in the α-glucan biosynthesis pathway, where it catalyzes the transfer of maltosyl units from M1P to elongating glucan chains. The integrity of this catalytic function appears to be tightly linked to bacterial survival. The observed conservation suggests that even minimal alterations in the amino acid sequence could have deleterious effects on enzyme activity, substrate binding, or structural stability. Given the enzyme's involvement in a pathway where substrate accumulation is intrinsically toxic, any impairment in GlgE function could disrupt metabolic balance. Importantly, previous studies have demonstrated that inhibition or inactivation of GlgE results in the intracellular accumulation of M1P, leading to a self-poisoning effect that rapidly kills the bacterium (Kalscheuer et al., 2010). In this context, the complete absence of mutations in *glgE* may reflect an evolutionary pressure to preserve enzymatic efficiency and prevent accumulation of toxic intermediates. Any mutation that reduces catalytic turnover or alters substrate processing could potentially trigger metabolic stress, thereby being strongly selected against during bacterial evolution.

The conservation of *glgE* across both regional clinical isolates and globally distributed strains also highlights its stability as a drug target. Genes that are highly conserved are less prone to developing resistance through mutational escape, making them particularly attractive candidates for therapeutic intervention. The lack of sequence variability suggests that inhibitors targeting GlgE are likely to retain efficacy across diverse *M.tb* lineages, reducing the risk of strain-specific variability in drug response. Overall, these findings provide strong evidence that *glgE* is under extreme evolutionary constraint and is essential for *M.tb* survival. The complete conservation of this gene across a large dataset reinforces its biological significance and supports its candidacy as a robust and universal drug target. This high degree of conservation, coupled with its unique metabolic role and susceptibility to substrate-induced toxicity, positions GlgE as a highly promising target for the development of next-generation anti-TB therapeutics.

### 3.2 Structure based virtual screening against GlgE active site led to the identification of twelve potential inhibitors

**Figure 1:**
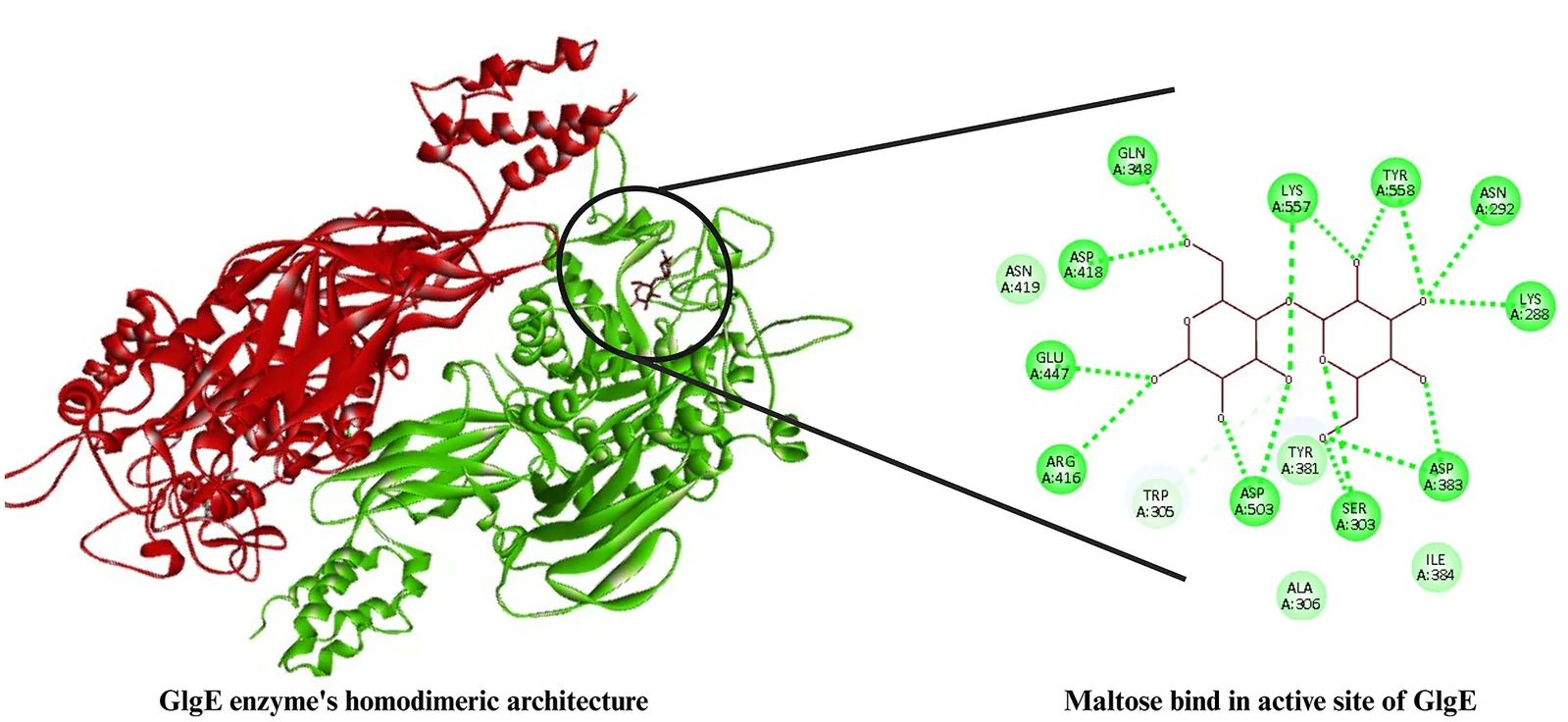
Three-dimensional structure and active-site architecture of the *M.tb* GlgE enzyme. **(Left)** Illustrates the homodimeric structure of GlgE, with the two monomers represented in red and green cartoon models. The catalytic pocket is highlighted within the circled region, indicating the substrate-binding cavity located at the interface of the catalytic domain. **(Right)** Shows a two-dimensional interaction map of maltose bound within the GlgE active site.

The catalytic site of GlgE is a highly conserved and structurally organized region that facilitates glycosyl transfer through a coordinated network of catalytic and substrate binding residues. At the core of the catalytic mechanism are Asp418, which acts as the nucleophile, and Glu447, which functions as the general acid residue as illustrated in Figure 1. These residues are essential for the cleavage and formation of glycosidic bonds during the transfer of maltosyl units. Surrounding the catalytic center, several residues contribute to substrate recognition and stabilization within the binding pocket. Asn419 plays a key role in maintaining substrate orientation through hydrogen bonding, while Tyr381 contributes to stabilization via stacking and polar interactions. Asp503 is involved in reinforcing substrate binding and maintaining the structural integrity of the catalytic environment. Additionally, Lys379 supports substrate positioning through electrostatic interactions, ensuring proper alignment for catalysis.

Within the subsite of the catalytic site, Ser303 is particularly important, as it forms hydrogen bonds with the glucosyl moiety of the substrate, aiding in its precise positioning. This interaction is critical for maintaining the correct orientation of M1P prior to catalysis as shown in Figure 1. Other residues such as Trp305 further contribute through hydrophobic and π-interactions, stabilizing the sugar moiety within the binding cleft. Collectively, the catalytic activity of GlgE depends on surrounding binding residues to ensure accurate substrate positioning and stabilization. This highly coordinated interaction network is essential for efficient enzymatic function and represents a key target region for inhibitor design. A grid was placed around this catalytic site encompassing all important residues.

The virtual screening workflow as described in the methods section, initially yielded a total of 48 hit compounds. These shortlisted molecules were re-ranked based on MM-GBSA score. To ensure structural diversity and to avoid redundancy, molecular fingerprint based clustering was performed, which resulted in the identification of 7 distinct clusters. Subsequent comprehensive analysis based on docking scores, MM-GBSA free energies and cluster representation led to prioritization of 12 top-ranking compounds as potential GlgE inhibitors as presented in Table 1. The chemical structures of VMP-1 to VMP-12 have been provided in the Supplementary Table 1. Additionally, the corresponding SMILES notation, molecular formula, and molecular weight for each compound are also provided.

**Table 1:** Binding affinity score and molecular interaction profiles of the top-ranked GlgE inhibitors identified by HTVS.

Sr. No.	Molecule	Docking score	XP score	Glide score	MMGBSA	Residues forming hydrogen bonds	Residues forming π Interactions
1	VMP-1	−12.229	−12.407	−12.407	−31.520	ASP503 ASN419 GLU447 TYR381 PHE449 SER303	TRP305 TYR558 LYS557
2	VMP-2	−12.859	−13.043	−13.043	−23.240	TYR381 ASP503 ARG416 ASP418 ASN419 GLU447	TRP305 Lys379
3	VMP-3	−12.169	−12.460	−12.460	−37.170	ASP383 GLN348	TYR381 Lys379 TRP305
4	VMP-4	−10.984	−10.989	−10.989	−27.400	ASP383	ILE384 TYR381 LYS380
5	VMP-5	−11.237	−11.457	−11.457	−34.38	GLN348 LYS380 LYS380	TYR381 TRP305 Lys379
6	VMP-6	−11.326	−12.180	−12.180	−62.830	ASP503 TYR381 ASN419	TRP305 PHE449
7	VMP-7	−11.408	−11.436	−11.436	−54.510	ASP418 SER303 ASP383 HIS421	TRP305 TYR381 PHE449 TRP472 LYS379
8	VMP-8	−11.533	−11.572	−11.572	−43.810	ASP418 GLU447 ASP503	TYR381 TYR558
9	VMP-9	−11.360	−11.389	−11.389	−51.620	GLU447 ASN419 HIS421 ASN376	
10	VMP-10	−11.553	−11.801	−11.801	−54.580	ASP418 LYS288 LYS380	TRP305 LYS379
11	VMP-11	−12.023	−12.023	−12.023	−31.810	GLU447 ASP503 TYR381 GLN348 SER303 LYS557	TRP305 TYR558 ILE384
12	VMP-12	−11.328	−11.328	−11.328	−36.490	SER303	TRP305 TYR381 LYS379 ILE384

These compounds exhibited favorable binding characteristics, as evidenced by their consistently strong docking scores and binding free energy values. The analysis revealed binding between the ligands and key residues within the GlgE active site. These interactions include hydrogen bonds, hydrophobic contacts and electrostatic interactions that facilitate precise ligand positioning and affinity. Collectively, these findings suggested that the identified molecules possessed significant potential as GlgE inhibitors providing a strong foundation for subsequent experimental validation.

Structure based molecular docking of the twelve shortlisted compounds (VMP-1 to VMP-12) revealed consistent and favorable binding within the catalytic pocket of GlgE, supporting their potential as enzyme inhibitors. Docking scores ranged from −10.98 to −12.86 kcal/mol, while MM-GBSA binding free energies varied from −23.24 to −62.83 kcal/mol, indicating strong protein–ligand interactions across the compound set. Among the evaluated molecules, VMP-6, VMP-7, VMP-9, and VMP-10 displayed the most favorable binding free energies (−62.83, −54.51, −51.62, and −54.58 kcal/mol, respectively), suggesting enhanced binding stability. Complete molecular docking scores for all shortlisted compounds are presented in Table 1.

Interaction analysis demonstrated that most compounds engaged residues critical for GlgE catalysis and substrate recognition. The catalytic residues Asp418 and Glu447 were frequently involved in hydrogen-bonding interactions, particularly in VMP-2, VMP-8, VMP-9, and VMP-10, indicating effective targeting of the core catalytic residues. Additional interactions with Asp503 and Asn419, residues implicated in substrate stabilization, were observed in several high-ranking compounds, further supporting their relevance to ligand binding.

Residues associated with substrate recognition, including Ser303, also contributed significantly to ligand binding. Compounds VMP-3, VMP-7, VMP-10, and VMP-12 interacted with Ser303, suggesting their ability to occupy the substrate-binding region and potentially interfere with M1P binding. Furthermore, aromatic residues such as Trp305, Tyr381, Phe449, and Tyr558 consistently participated in π-mediated and hydrophobic interactions, providing substantial stabilization of the protein–ligand complexes.

Among all compounds, VMP-6 emerged as the strongest candidate, exhibiting the lowest MM-GBSA energy and forming interactions with Asp503, Asn419, Tyr381, Trp305, and Phe449. VMP-7 also demonstrated an extensive interaction network involving catalytic, substrate-binding, and aromatic residues, while VMP-9 and VMP-10 combined favorable binding energies with strong hydrogen-bonding and hydrophobic contacts. Compounds VMP-1, VMP-2, VMP-3 and VMP-5 likewise showed robust interaction profiles characterized by multiple hydrogen bonds and aromatic contacts. In contrast, VMP-4 displayed comparatively weaker binding, although their interactions suggest potential for further structural optimization. VMP-8, VMP-11, and VMP-12 exhibited intermediate binding characteristics while maintaining interactions with key active-site residues as depicted in Table 2.

**Table 2:** Two-dimensional interaction profiles and active-site binding modes of compounds VMP-1 to VMP-12 within the GlgE catalytic pocket.

Compound	Two-dimensional interaction	Active-site binding representation
VMP-1	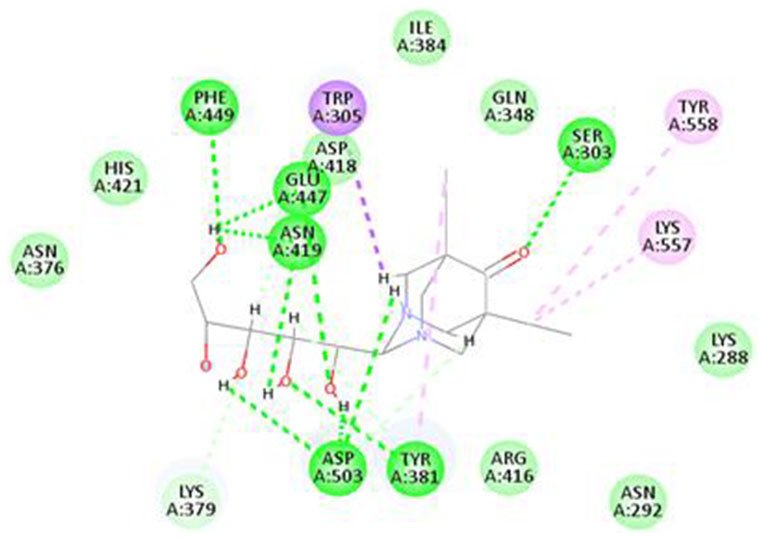	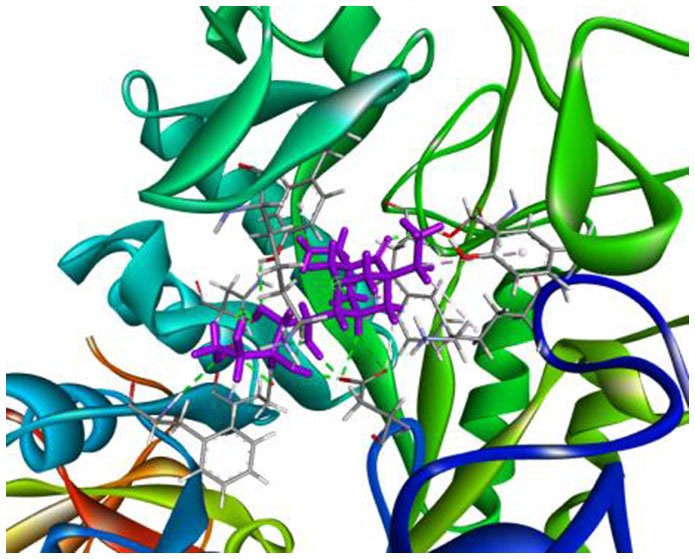
VMP-2	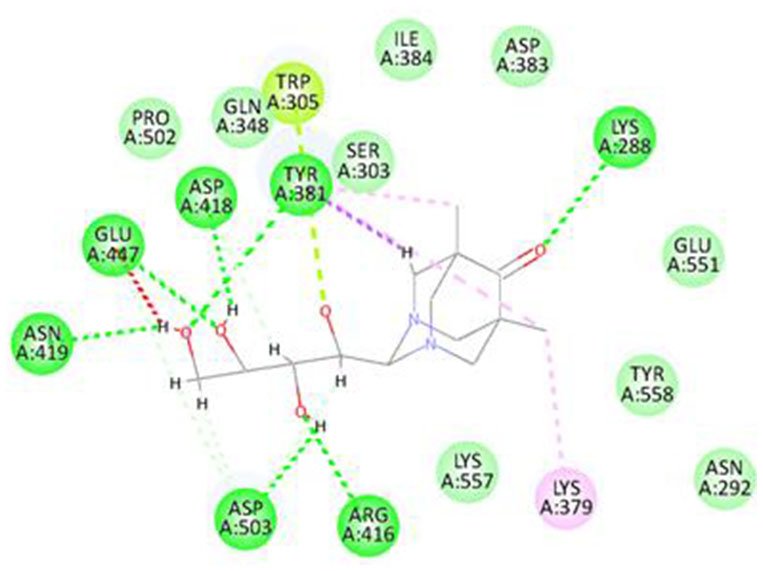	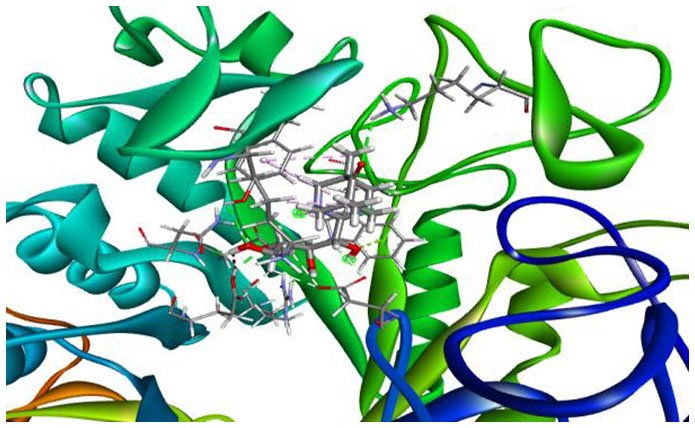
VMP-3	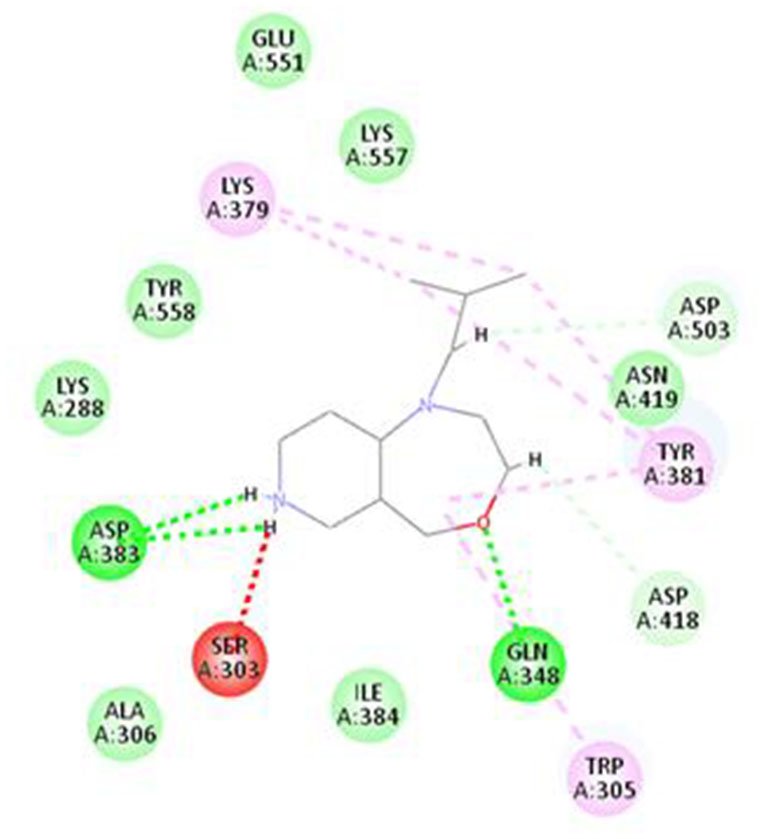	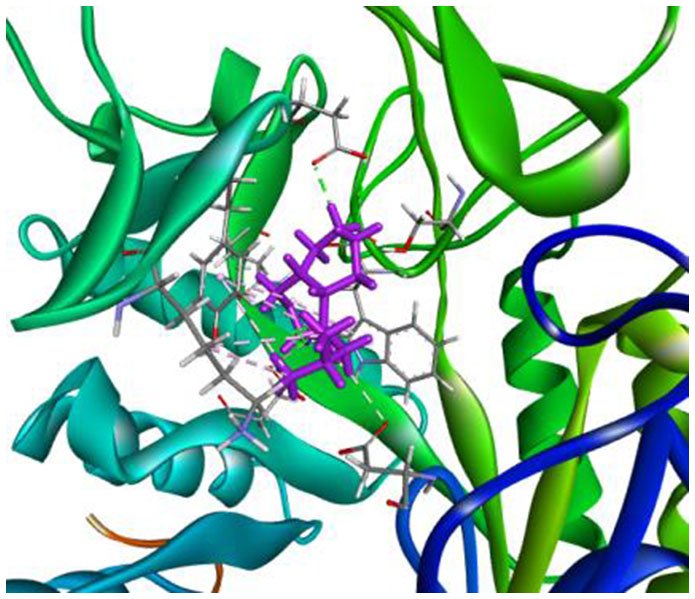
VMP-4	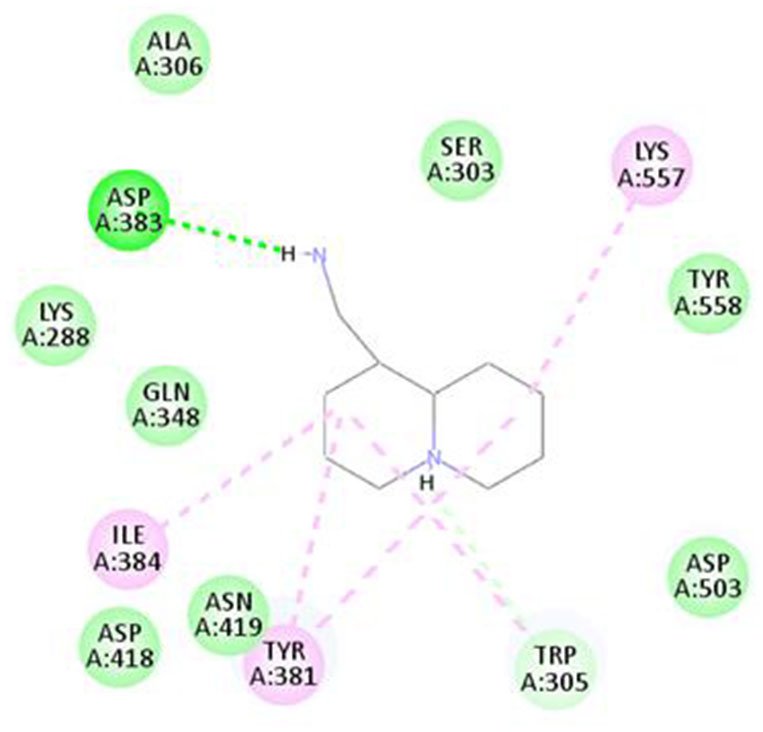	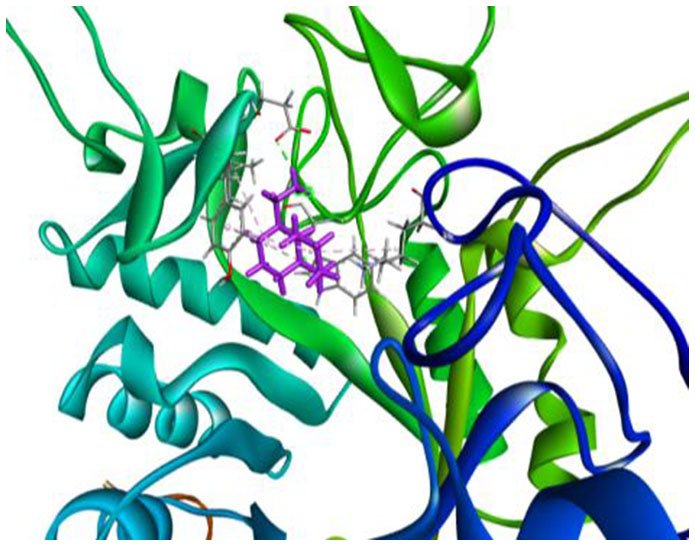
VMP-5	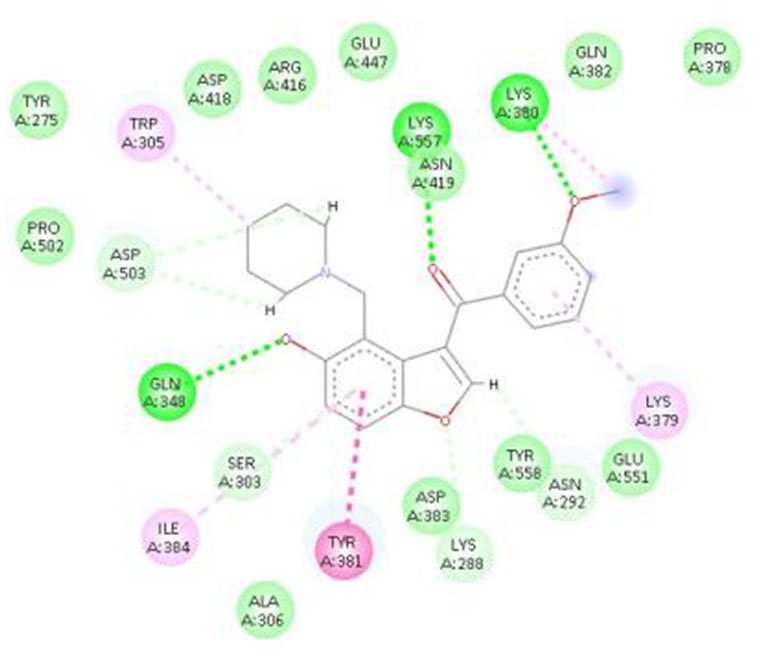	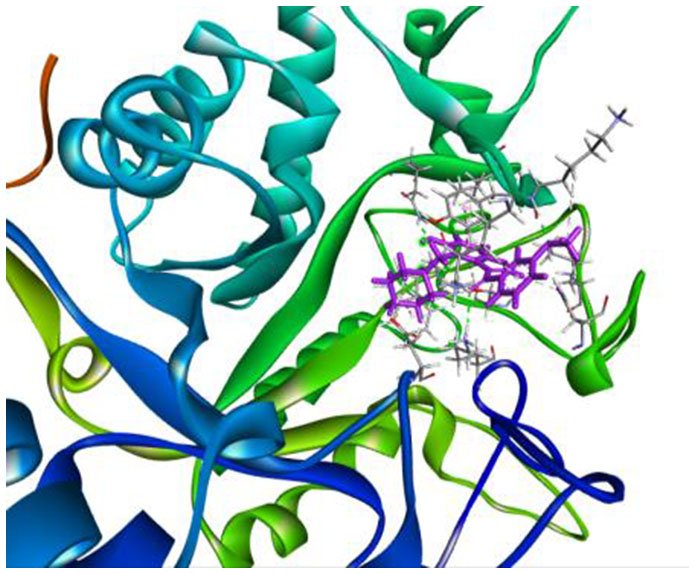
VMP-6	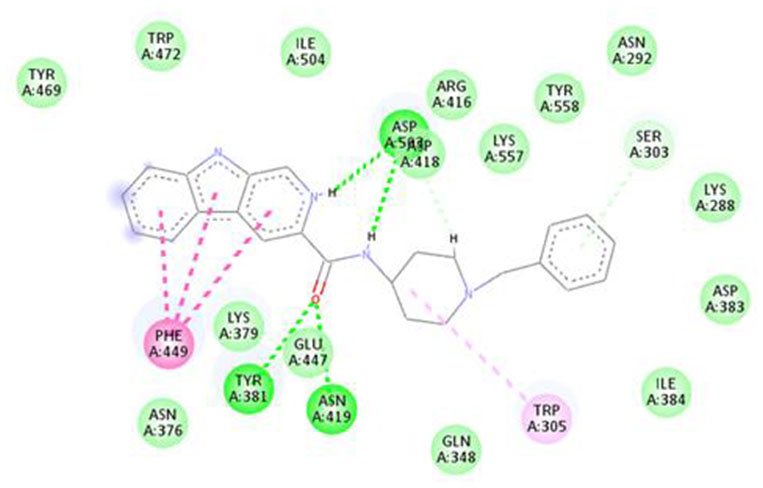	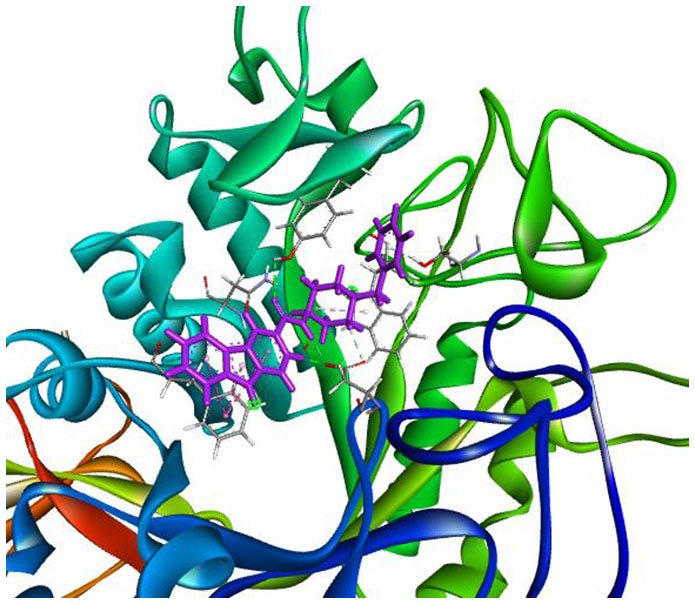
VMP-7	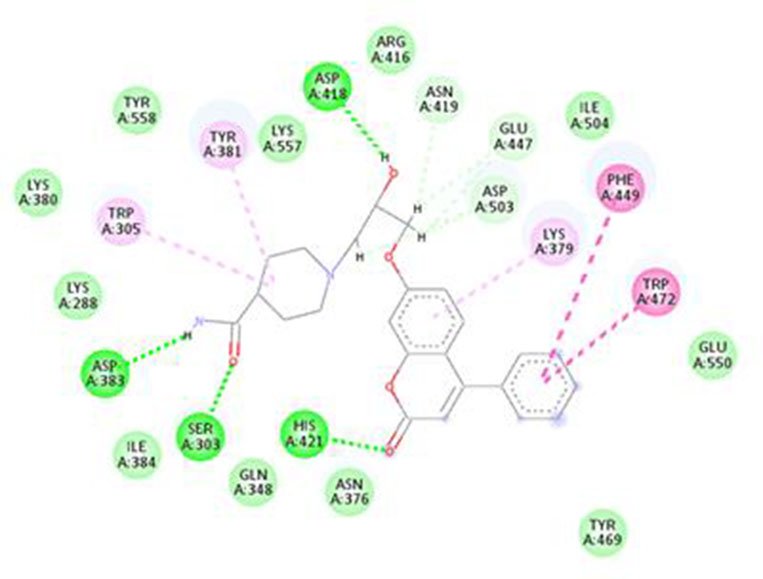	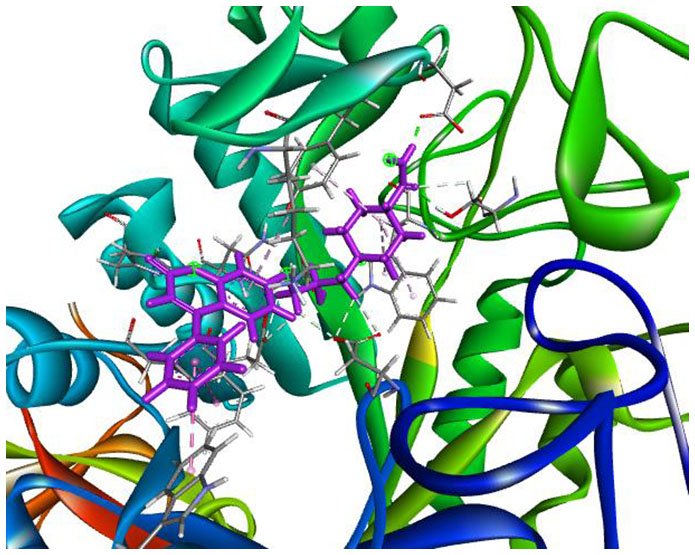
VMP-8	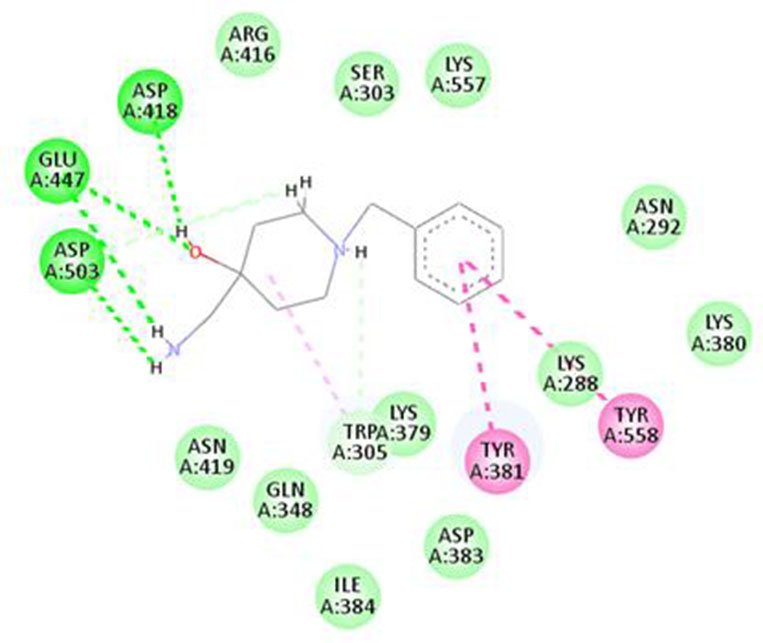	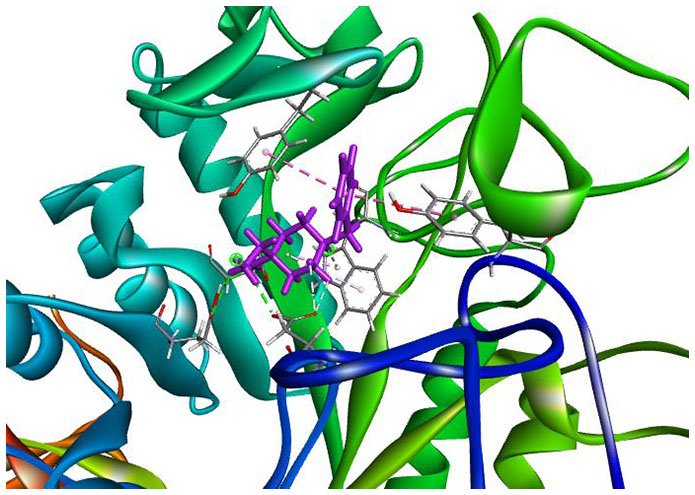
VMP-9	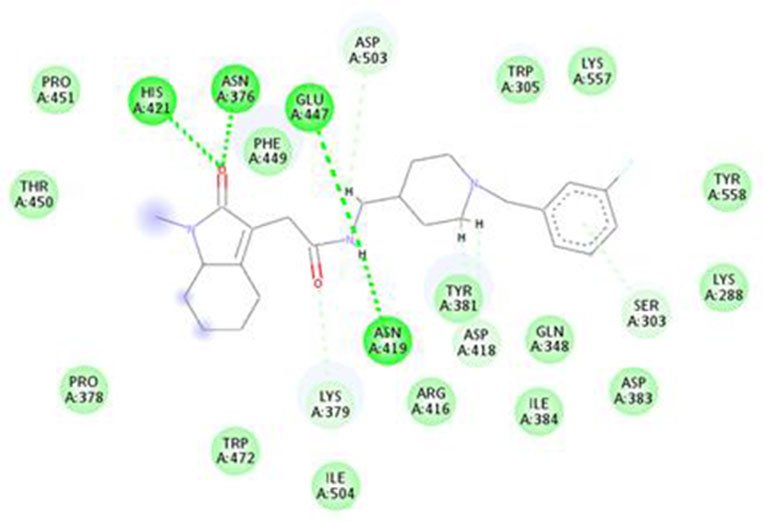	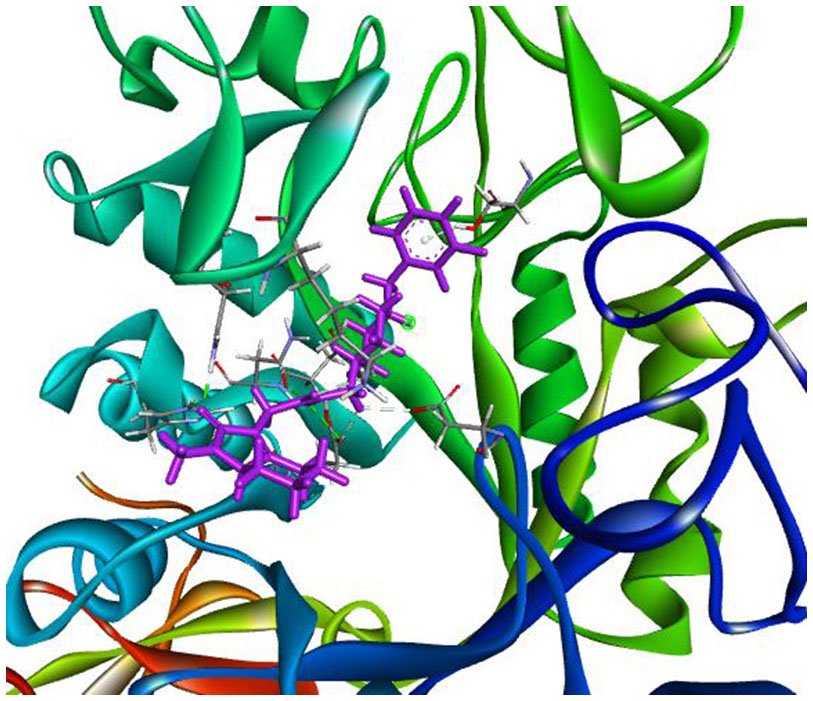
VMP-10	i 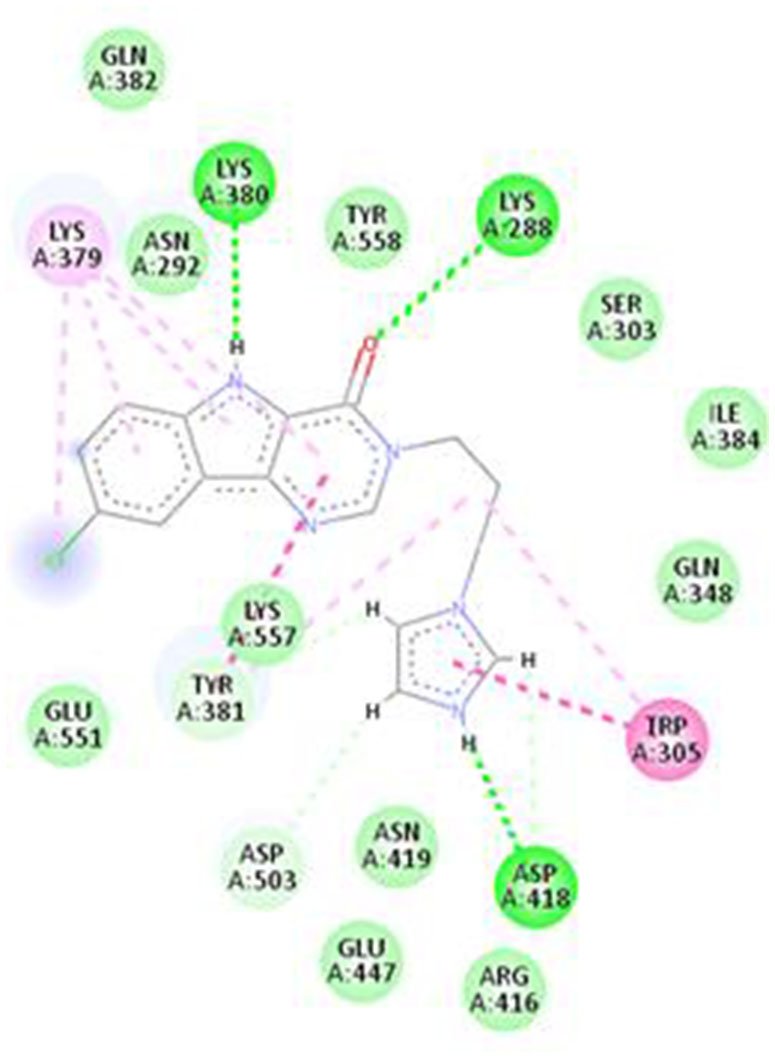	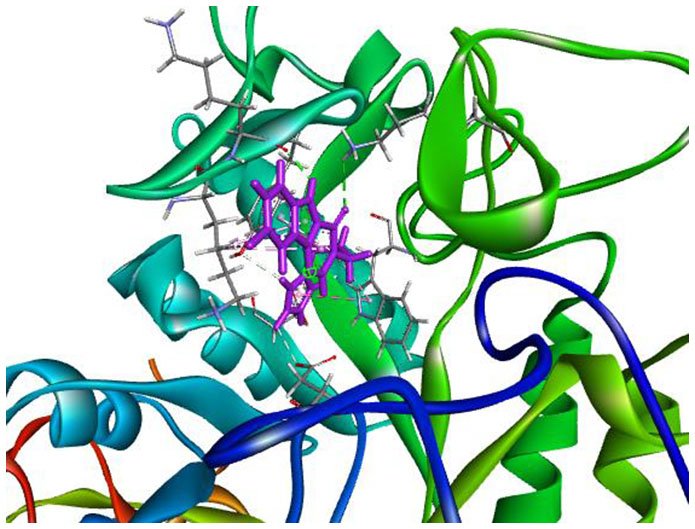
VMP-11	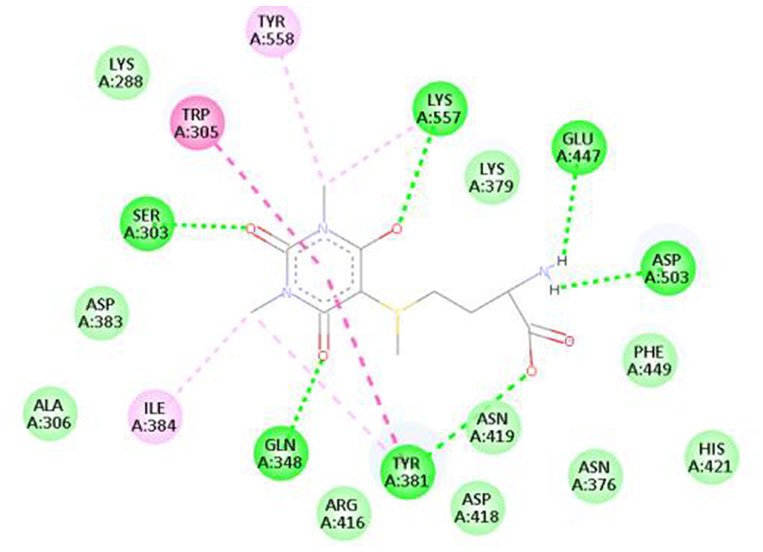	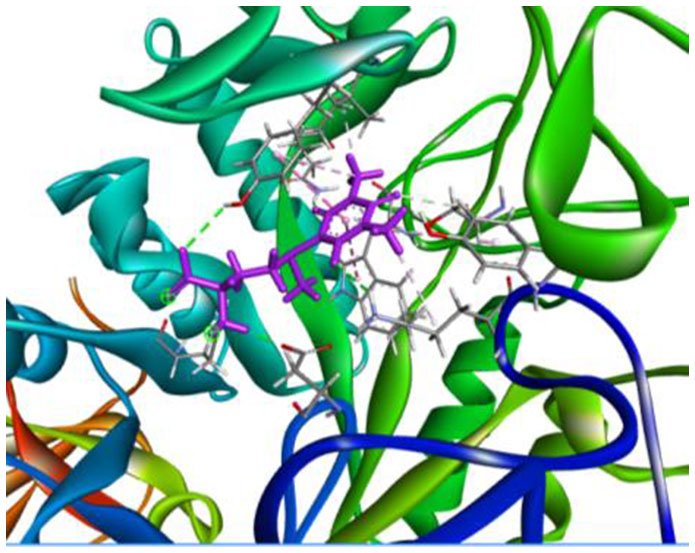
VMP-12	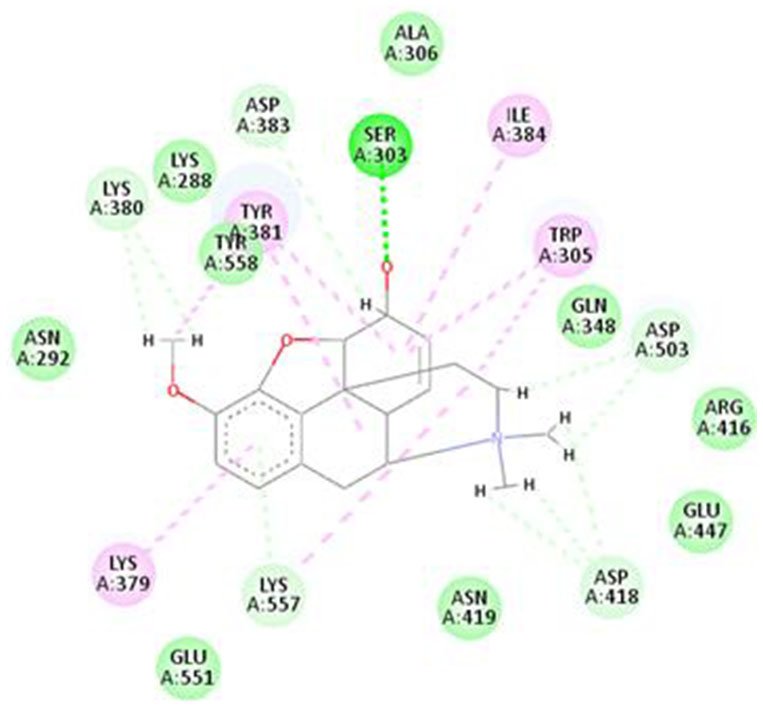	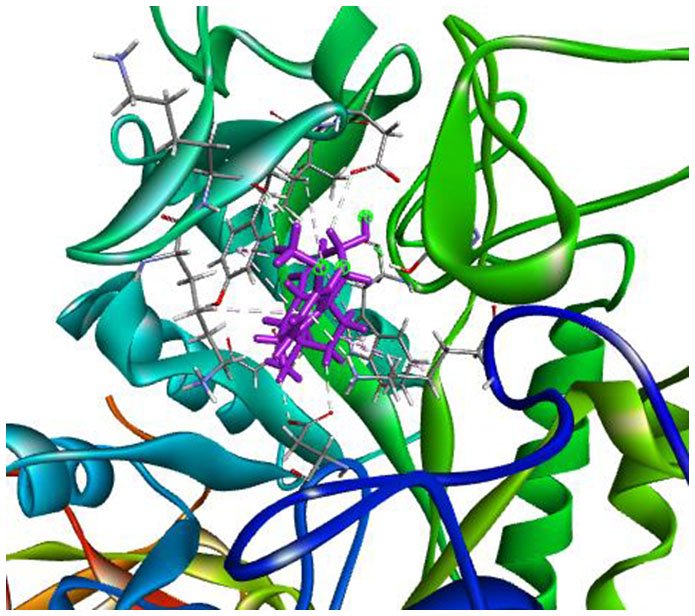

Collectively, all twelve compounds shared a conserved interaction pattern involving catalytic residues (Asp418 and Glu447), substrate-binding residues (Asn419, Asp503, and Ser303), and aromatic residues (Trp305, Tyr381, and Phe449). The ability of several compounds to simultaneously engage these functional regions suggests strong binding specificity and supports their proposed mechanism as competitive inhibitors of GlgE. These findings identify VMP-6, VMP-7, VMP-9, and VMP-10 as the most promising candidates and provide a strong structural foundation for subsequent experimental validation and anti-TB drug development.

### 3.3 VMP-7 emerges as a lead GlgE inhibitor with significant anti-mycobacterial activity against *M.tb*

The anti-mycobacterial activity of the twelve GlgE-targeting compounds (VMP-1 to VMP-12) was evaluated against *M.tb* using a resazurin-based viability assay at concentrations of 50, 125, and 250 μg/mL.

**Figure 2:**
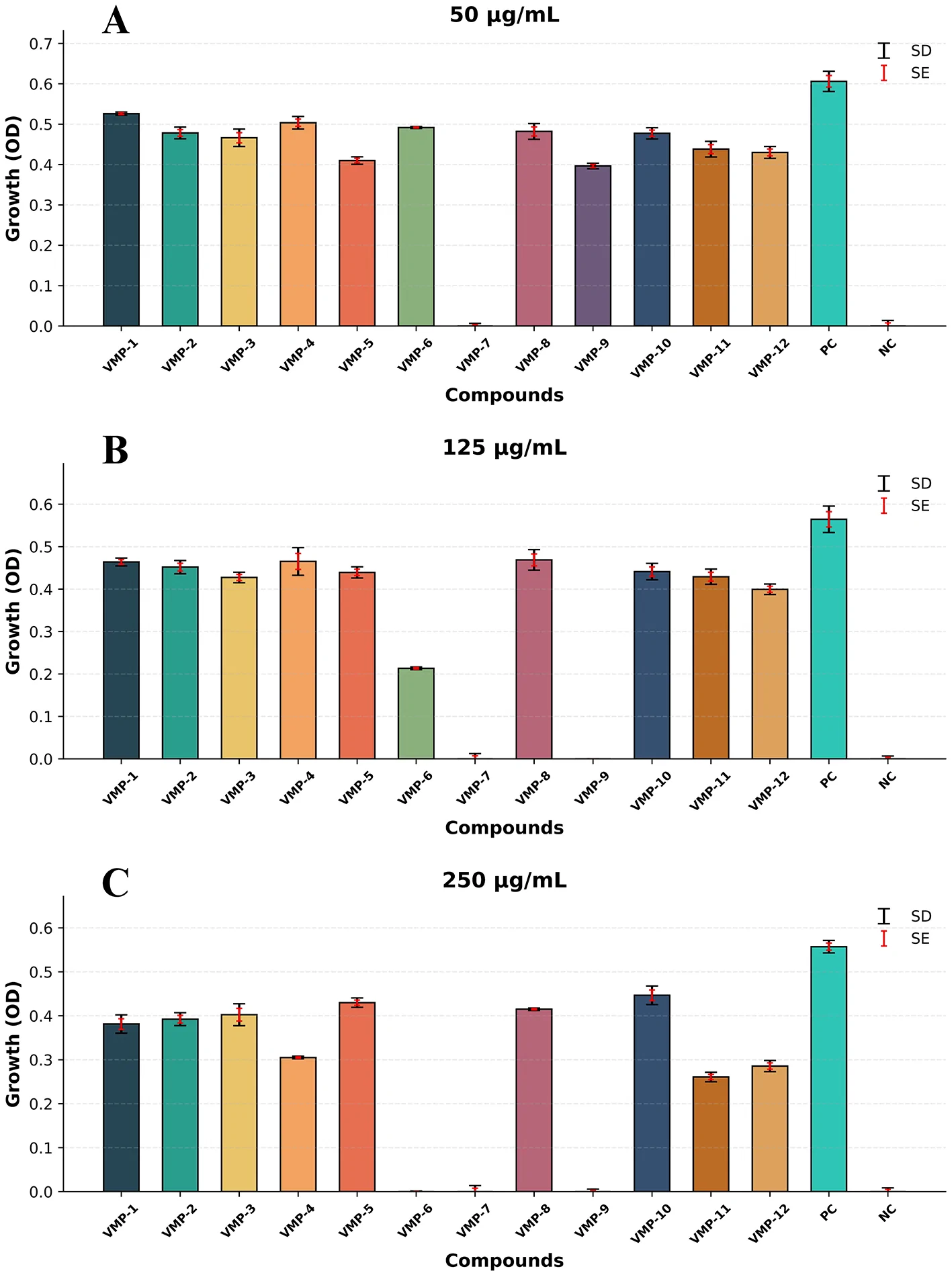
Screening of GlgE targeting compounds (VMP-1 to VMP-12) for anti-mycobacterial activity against *M.tb* using a resazurin-based viability assay. Bacterial growth was assessed by optical density (OD) measurements following treatment with the indicated compounds at 50 μg/mL **(A)**, 125 μg/mL **(B)**, 250 μg/mL **(C)**, Positive control (PC) is untreated bacterial culture and Negative control (NC) is only sterile media.

A clear dose-dependent inhibitory trend was observed for most compounds, with growth inhibition generally increasing as compound concentration increased. Among all candidates, VMP-7 demonstrated the strongest and most consistent anti-mycobacterial activity, maintaining near-complete growth inhibition across all tested concentrations, including the lowest concentration of 50 μg/mL as illustrated in Figure 2.

This exceptional potency suggests a robust inhibitory effect on bacterial viability and identifies VMP-7 as the most promising lead compound in the series. VMP-6 and VMP-9 also exhibited substantial growth inhibition at higher concentrations, particularly at 250 μg/mL, but their activity decreased progressively upon dilution, indicating a concentration-dependent effect. The remaining compounds displayed moderate to weak inhibitory activity. VMP-1, VMP-2, VMP-3, VMP-10, VMP-11, and VMP-12 showed partial growth suppression, whereas VMP-4 and VMP-5 were among the least active compounds across the tested concentration range. Although several compounds retained measurable activity at intermediate concentrations, only VMP-7 consistently maintained strong inhibition independent of dose.

Overall, the assay revealed marked differences in anti-mycobacterial efficacy among the shortlisted GlgE inhibitors. The superior and sustained activity of VMP-7, coupled with the concentration-dependent effects observed for VMP-6 and VMP-9, highlights these compounds as the most promising candidates for further investigation. These findings provide biological validation of the structure-based screening approach and support the potential of GlgE as a viable target for anti-TB drug development.

### 3.4 VMP-7 exhibits a minimum inhibitory concentration of 12.5 μg/mL against *M.tb*

Among the twelve GlgE-targeting compounds (VMP-1 to VMP-12) screened using the resazurin-based viability assay, VMP-7 emerged as the most promising candidate due to its pronounced and reproducible concentration-dependent inhibition of *M.tb* growth. The remaining compounds exhibited limited antibacterial activity and maintained relatively high OD values across the tested concentrations, indicating minimal growth suppression. VMP-7 demonstrated substantial inhibition of bacterial growth, achieving complete suppression at 50 μg/mL. Based on its superior antimycobacterial activity and consistent performance across replicates, VMP-7 was selected for further MIC determination.

**Figure 3:**
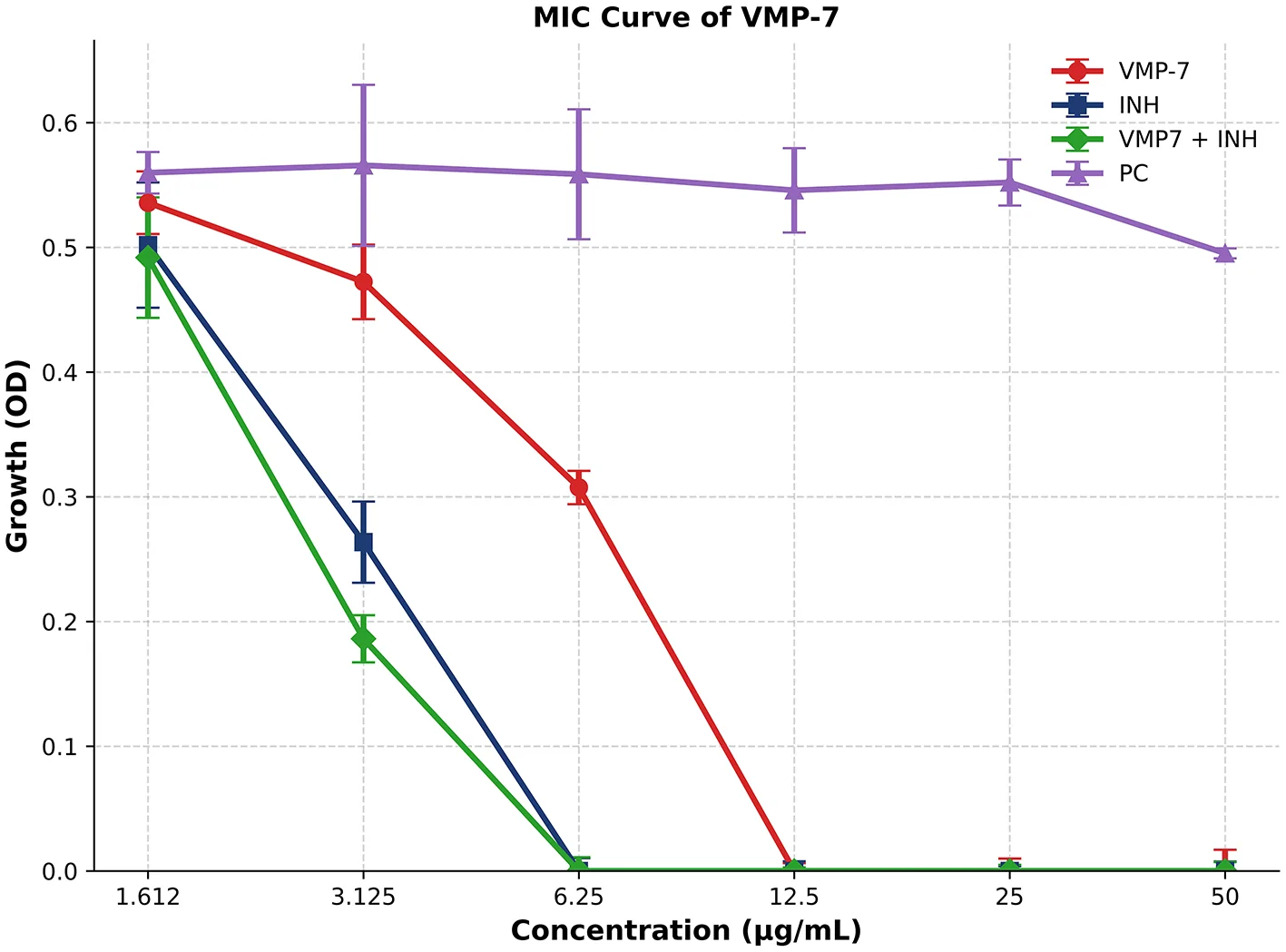
Determination of the MIC of VMP-7 against *M.tb* using a resazurin-based viability assay. Bacterial growth was assessed by optical density (OD) measurements following treatment with increasing concentrations of VMP-7 (1.612 to 50 μg/mL), INH, and a combination of VMP-7 with INH. Positive control (PC) is untreated bacterial culture.

The MIC of VMP-7, both alone and in combination with INH, was evaluated using the same resazurin-based viability assay. The untreated positive control maintained consistently high OD values throughout the experiment, confirming sustained bacterial growth and validating the robustness of the assay. INH exhibited a characteristic dose-dependent inhibitory profile, with a marked reduction in bacterial growth observed between 3.125 and 6.25 μg/mL. Complete inhibition was achieved at 6.25 μg/mL, consistent with its established antimycobacterial activity as illustrated in Figure 3.

VMP-7 also displayed concentration-dependent inhibition, although with a more gradual response. Minimal inhibition was observed at 1.612 μg/mL, while progressive reductions in bacterial growth were evident at 3.125 and 6.25 μg/mL. Complete growth inhibition was achieved at 12.5 μg/mL, establishing the MIC of VMP-7 under the tested conditions. the combination of VMP-7 and INH demonstrated enhanced antibacterial efficacy relative to either agent alone. While limited effects were observed at the lowest concentration tested, a pronounced reduction in bacterial growth was evident at 3.125 μg/mL. Complete inhibition was achieved at 6.25 μg/mL. Overall, MIC analysis established that VMP-7 possesses significant antimycobacterial activity with an MIC of 12.5 μg/mL. These findings identify VMP-7 as a promising GlgE-targeting lead compound and support its further evaluation as a potential adjunct therapeutic agent for TB treatment.

### 3.5 VMP-7 demonstrates low cytotoxicity effective against THP-1 derived macrophages

The cytotoxicity of VMP-7 was evaluated in THP-1-derived macrophages using the MTT assay following exposure to concentrations ranging from 25 to 800 μg/mL for 24, 48, and 72 h. VMP-7 exhibited a clear concentration- and time-dependent effect on cell viability, with minimal toxicity at lower concentrations and progressive reductions in viability at higher doses.

**Figure 4:**
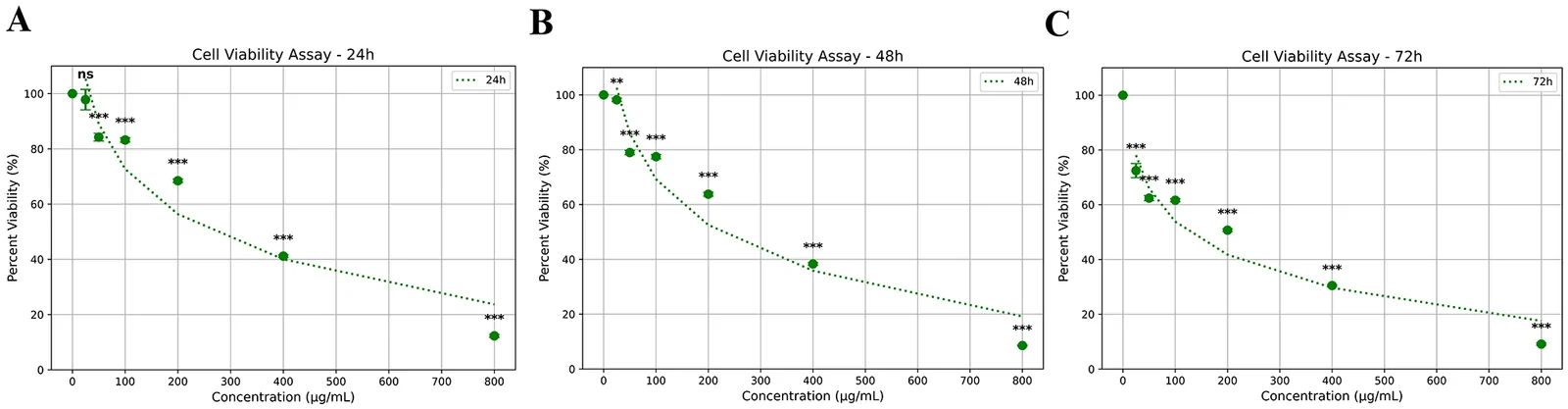
Cytotoxicity assessment of VMP-7 in THP-1-derived macrophages using the MTT assay. Cell viability was determined following exposure to increasing concentrations of VMP-7 (0–800 μg/mL) for **(A)** 24 h, **(B)** 48 h, and **(C)** 72 h. Standard derivation and statistical significance was determined using an unpaired Student's t-test, and significance levels are indicated as follows: ns, not significant (*P* ≥ 0.05); *P < 0.05; **P < 0.01; ***P < 0.001.

At 24 h, THP-1 derived macrophages showed high tolerance to VMP-7 at concentrations between 25 and 100 μg/mL, maintaining cell viability above 80%. Cytotoxic effects became evident at 200 μg/mL, where viability decreased to approximately 70%. A more pronounced reduction was observed at 400 μg/mL, with viability declining to 40%, while the highest concentration tested (800 μg/mL) reduced viability to approximately 10%, indicating severe cytotoxicity as illustrated in Figure 4A.

A similar dose-dependent trend was observed at 48 h. Cell viability remained high (>85%) at concentrations up to 100 μg/mL, indicating sustained cellular tolerance. However, viability decreased to ~65% at 200 μg/mL and further declined to 40% and 10% at 400 and 800 μg/mL, respectively as illustrated in Figure 4B. These findings suggest increased cellular sensitivity following prolonged exposure.

At 72 h, the cytotoxic effects of VMP-7 became more pronounced. Viability remained relatively preserved at lower concentrations, with approximately 70–75% viable cells observed between 25 and 100 μg/mL. In contrast, viability decreased to 50% at 200 μg/mL and further declined to approximately 30% at 400 μg/mL. Consistent with earlier time points, exposure to 800 μg/mL resulted in severe toxicity, reducing viability to approximately 10% as illustrated in Figure 4C.

Overall, VMP-7 demonstrated a concentration-dependent reduction in macrophage viability, with minimal cytotoxicity observed at concentrations between 25 and 100 μg/mL and substantial toxicity at concentrations ≥400 μg/mL. Time-course analysis further indicated that prolonged exposure enhanced the cytotoxic response, particularly at intermediate and higher concentrations. Importantly, the concentration range associated with significant antimycobacterial activity (25–50 μg/mL) maintained high macrophage viability (>90%), indicating a favorable therapeutic window. These findings establish that VMP-7 possesses a well-defined safety profile, exhibiting low toxicity at therapeutically relevant concentrations while inducing significant cytotoxic effects only at substantially higher doses. Collectively, the results support the further development of VMP-7 as a potential anti-TB candidate with acceptable host-cell tolerability.

### 3.6 VMP-7 reduces intracellular survival of drug-sensitive and drug resistances *M.tb*

The intracellular antimycobacterial activity of VMP-7 was evaluated using a THP-1-derived macrophages infection model against both drug-sensitive *M.tb* H37Rv and a MDR clinical isolate. Macrophages were treated with VMP-7 at 25 μg/mL (U25) and 50 μg/mL (U50), either alone or in combination with standard anti-TB drugs. Bacterial survival was assessed by CFU enumeration at 48 h and 72 h post-treatment.

**Figure 5:**
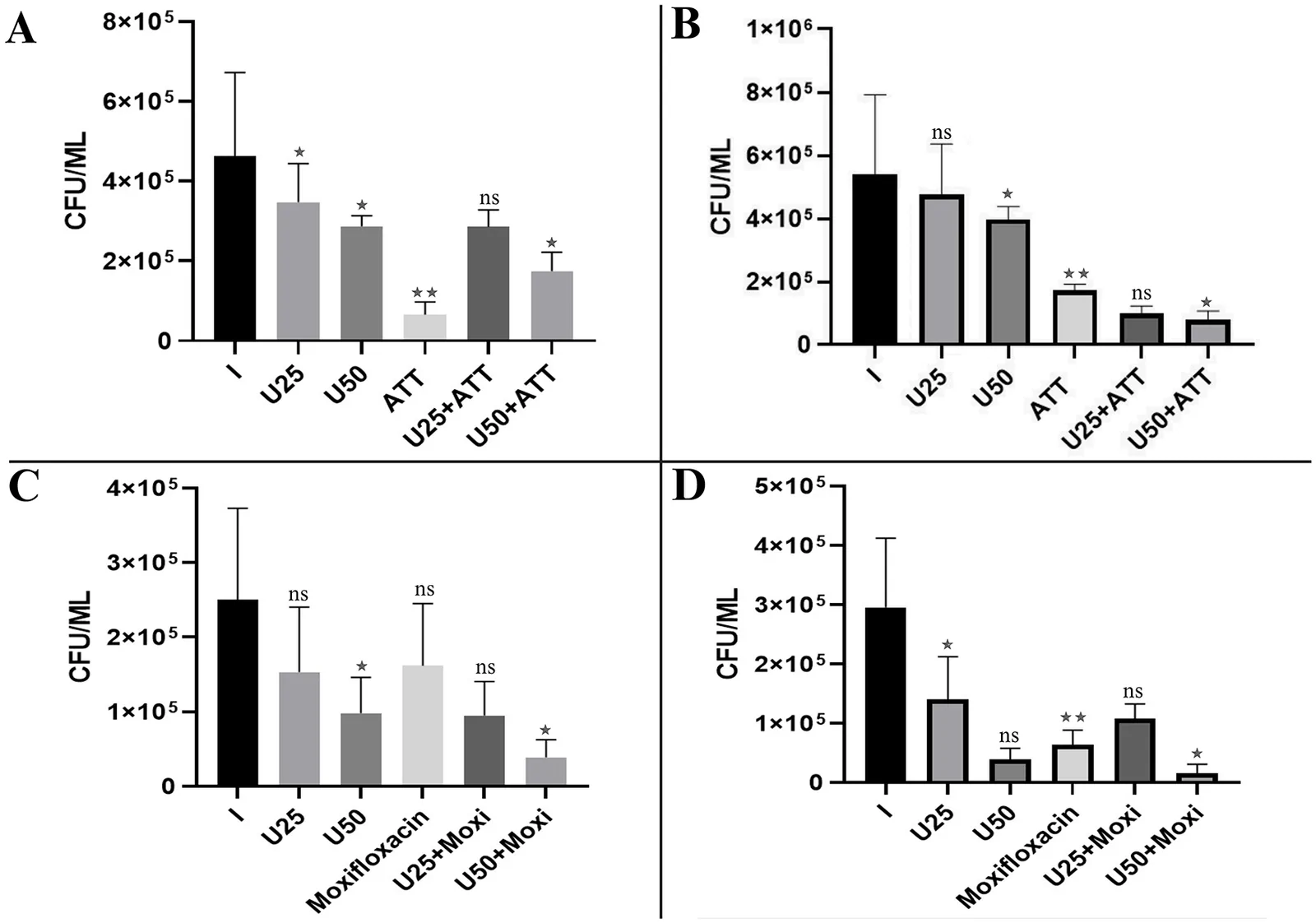
Intracellular anti-mycobacterial activity of VMP-7 against drug-sensitive and MDR *M.tb* in THP-1 derived macrophages. Intracellular bacterial survival was quantified by CFU enumeration following treatment with VMP-7 at 25 μg/mL (U25) and 50 μg/mL (U50), either alone or in combination with standard anti-TB drugs, “I” denote the positive growth control. **(A)** Drug-sensitive *M.tb* H37Rv after 48 h treatment with VMP-7, anti-TB therapy (ATT; isoniazid + rifampicin). **(B)** *M.tb* H37Rv after 72 h treatment VMP-7, ATT therapy. **(C)** MDR *M.tb* clinical isolate after 48 h treatment with VMP-7, moxifloxacin (2 μg/mL). **(D)** MDR isolate after 72 h treatment with VMP-7 and moxifloxacin combination treatments. Standard deviation and statistical significance was determined using an unpaired *t*-test, and significance levels are indicated as follows: ns, not significant (*P* ≥ 0.05); *P < 0.05; **P < 0.01 and ***P < 0.001.

In H37Rv infected macrophages, untreated controls maintained a high intracellular bacterial burden (~5 × 10^5^ CFU/mL) throughout the study, indicating active bacterial replication. At 48 h, treatment with VMP-7 resulted in a concentration-dependent reduction in intracellular bacterial survival. U25 reduced the bacterial burden to approximately 3.5 × 10^5^ CFU/mL, whereas U50 further reduced CFU counts to 2.6 × 10^5^ CFU/mL. Combination treatment further enhanced bacterial clearance, with U25+ATT and U50+ATT reducing bacterial loads to ~2.5 × 10^5^ and 1.8 × 10^5^ CFU/mL, respectively as illustrated in Figure 5A.

A more pronounced reduction in intracellular bacterial burden was observed after 72 h of treatment. While untreated controls remained at approximately 5 × 10^5^ CFU/mL, VMP-7 treatment resulted in further decreases in bacterial survival. U50 demonstrated greater efficacy than U25, confirming a dose-dependent response. The standard drug treatment reduced bacterial counts to approximately 2 × 10^5^ CFU/mL, whereas combination treatments produced the strongest antibacterial effect. U25+ATT reduced bacterial counts to approximately 1 × 10^5^ CFU/mL, while U50+ATT reduced the bacterial burden to 0.5 × 10^5^ CFU/mL, approaching the detection limit of the assay as illustrated in Figure 5B. Collectively, these findings demonstrate that VMP-7 possesses significant intracellular activity against drug-sensitive *M.tb*, which is enhanced by prolonged exposure and combination treatment.

The activity of VMP-7 was subsequently evaluated against an MDR clinical isolate using moxifloxacin (2 μg/mL) as the reference drug. At 48 h, untreated macrophages exhibited an intracellular bacterial burden of approximately 2.5 × 10^5^ CFU/mL. Treatment with U25 reduced bacterial counts to 1.5 × 10^5^ CFU/mL, whereas U50 further reduced the burden to 0.9 × 10^5^ CFU/mL, indicating concentration-dependent activity against drug-resistant bacteria. Moxifloxacin treatment resulted in bacterial counts of approximately 1.5 × 10^5^ CFU/mL. Combination treatments produced enhanced bacterial clearance, with U25+moxifloxacin and U50+moxifloxacin reducing bacterial loads to approximately 0.8 × 10^5^ and 0.3 × 10^5^ CFU/mL, respectively as illustrated in Figure 5C.

At 72 h, untreated MDR-infected macrophages showed a bacterial burden of approximately 3 × 10^5^ CFU/mL. VMP-7 treatment resulted in further reductions in bacterial survival, with U50 demonstrating substantially greater activity than U25. Moxifloxacin alone reduced bacterial counts to ~0.5 × 10^5^ CFU/mL, whereas the combination treatments exhibited the highest efficacy. U25+moxifloxacin reduced bacterial counts to ~1 × 10^5^ CFU/mL, while U50+moxifloxacin reduced the burden to 0.1 × 10^5^ CFU/mL, indicating near-complete intracellular bacterial clearance as illustrated in Figure 5D.

To quantitatively assess the interaction between the VMP-7 and standard anti-TB drugs, the quantitative synergy index (SI) was calculated using the Loewe additivity model based on normalized bacterial survival (Lederer et al., 2018). In the H37Rv infected macrophage at 48 h, the combinations of U25 and ATT (SI = 5.9) and U50 and ATT (SI = 4.4) exhibited antagonistic interactions, indicating reduced bactericidal activity compared with the expected additive effect. However, after 72 h of treatment, the interaction shifted markedly toward synergy, with SI values of 0.56 and 0.51 for the U25+ATT and U50+ATT combinations, respectively, demonstrating enhanced intracellular killing following prolonged exposure. In the MDR *M.tb* model, the U25+moxifloxacin combination displayed an additive interaction at 48 h (SI = 1.0), whereas the U50+moxifloxacin combination showed moderate synergy (SI = 0.63). For 72 h, U25+moxifloxacin combinations exhibited antagonistic interactions, with SI values of 3.4 and for U50+moxifloxacin moderate synergy with SI values of 0.9. Collectively, these findings demonstrate that the interaction between the VMP-7 and standard anti-TB drugs is time-dependent. While prolonged treatment enhanced synergistic activity against the drug-susceptible H37Rv strain, the MDR isolate exhibited moderate synergy efficacy.

Overall, VMP-7 exhibited significant dose- and time-dependent intracellular antimycobacterial activity against both drug sensitive and MDR *M.tb* strains. Enhanced bacterial clearance was consistently observed with combination therapy, supporting the potential utility of VMP-7 as an adjunct anti-TB agent. Furthermore, the comparable activity observed against both H37Rv and MDR strains highlights its potential for the treatment of drug-resistant TB.

## 4 Discussion

The present study was designed to address a central challenge in TB drug discovery. The identification of novel, mechanistically distinct targets and inhibitors capable of overcoming the growing burden of drug resistant *M.tb*. By integrating large scale genomic conservation analysis, structure based virtual screening, and multi-tiered experimental validation, this work provides both conceptual and practical insights into targeting the GlgE mediated α-glucan biosynthesis pathway. Importantly, beyond identifying lead compounds, the study interrogates the biological robustness of the target itself, an aspect often overlooked in early-stage drug discovery.

One of the most striking findings is the complete sequence conservation of the *glgE* gene across 382 geographically and genetically diverse isolates. While essential genes are generally conserved, the absence of even synonymous substitutions suggests an unusually high degree of evolutionary constraint. This observation supports the hypothesis that GlgE operates within a finely balanced metabolic framework, where even minimal perturbations may compromise enzyme function. Mechanistically, this can be rationalized by the “self-poisoning” phenomenon associated with GlgE inhibition, whereby accumulation of M1P induces toxic metabolic stress (Kalscheuer et al., 2010). The lack of sequence variability may therefore reflect strong purifying selection against mutations that could destabilize catalytic efficiency or substrate processing. From a drug discovery perspective, such invariance is highly desirable, as it suggests a reduced propensity for resistance via target mutation. However, this assumption warrants caution: resistance mechanisms in *M.tb* are often multifactorial and may arise through compensatory metabolic adaptations, efflux systems, or pathway redundancy rather than direct target mutation (Nguyen, 2016). Thus, while *glgE* conservation strengthens its candidacy as a drug target, it does not preclude the possibility of resistance emerging through indirect mechanisms.

The structure based virtual screening approach employed in this study successfully identified a set of chemically diverse compounds that engage the GlgE active site. A key strength of the docking results lies in the convergence of interaction patterns across independent compounds, particularly the recurrent engagement of catalytic residues Asp418 and Glu447, along with substrate-stabilizing residues such as Asn419, Asp503, and Ser303. This convergence suggests that the docking protocol effectively captured biologically relevant binding modes. Moreover, the extensive involvement of aromatic residues Trp305 and Tyr381 highlights the importance of hydrophobic and π-mediated interactions in stabilizing ligand binding within the catalytic pocket.

Despite these strengths, it is important to recognize the inherent limitations of docking-based predictions. Binding affinity estimates derived from scoring functions and MM-GBSA calculations provide only an approximation of true thermodynamic behavior. Therefore, while the identification of high-affinity candidates such as VMP-6, VMP-7, VMP-9, and VMP-10 is encouraging, these findings should be interpreted as prioritization rather than definitive evidence of binding efficacy as is revealed by further experimentation.

Among the identified compounds, VMP-7 emerged as the most promising candidate based on its experimental performance. Notably, while several compounds exhibited strong docking scores, only a subset translated into measurable biological activity, underscoring a common disconnect between in silico predictions and *in vitro* efficacy. This discrepancy highlights the importance of experimental validation and suggests that factors such as membrane permeability, intracellular accumulation, and metabolic stability play critical roles in determining compound efficacy preliminary due to limitations of docking tools.

The resazurin-based viability assay revealed a clear concentration-dependent inhibitory profile, with VMP-7 demonstrating superior and consistent activity compared to other selected candidates. However, the observed MIC of ~12.5 μg/mL indicates moderate potency relative to established first-line drugs such as INH. This raises important considerations regarding its therapeutic potential as a standalone agent.

The intracellular infection model provides an additional layer of validation, demonstrating that VMP-7 retains activity within THP-1 derived macrophages, the primary niche of *M.tb*. The observed dose and time-dependent reduction in bacterial burden is particularly significant, as intracellular efficacy is often a major bottleneck in TB drug development. The enhanced activity observed in combination treatments further supports the potential of VMP-7 as part of a multi-drug regimen. Importantly, the compound also exhibited activity against multidrug-resistant strains, suggesting that its mechanism of action is distinct from those of conventional antibiotics. The cytotoxicity profile of VMP-7 indicates a favorable therapeutic window, with minimal toxicity observed at concentrations relevant for antimicrobial activity.

Although VMP-7 exhibited a higher MIC (12.5 μg/mL) than the first-line anti-TB drugs, its activity should be interpreted in the context of its identification as an early-stage lead compound. The relatively modest potency highlights the need for further medicinal chemistry optimization through systematic structure activity relationship studies. As a limitation of the present study, direct experimental evidence confirming VMP-7 binding to GlgE, inhibition of GlgE enzymatic activity, and consequent metabolic poisoning through M1P accumulation was not established. Further studies involving *in vivo* efficacy, pharmacokinetic/pharmacodynamic profiling, and animal-model validation will be required to determine the therapeutic potential of VMP-7 as an anti-TB drug candidate. Additionally, it would be valuable to demonstrate the disruption of cellular homeostasis and cell wall-associated processes using advanced imaging techniques such as electron microscopy.

Our study provides an important proof-of-concept for targeting the GlgE pathway in *M.tb*. The identification of VMP-7 as a lead compound, combined with its demonstrated intracellular activity and synergistic potential, highlights the feasibility of exploiting metabolic vulnerabilities in *M.tb*. More broadly, the work underscores the value of integrating genomic, structural, and experimental approaches in drug discovery. Such strategies are particularly critical in the context of TB, where the complexity of the pathogen and the emergence of resistance demand innovative and multidisciplinary solutions.

## 5 Conclusion

This study identifies GlgE as a highly conserved and therapeutically relevant drug target in *M.tb*. Sequence analysis revealed complete conservation of the *glgE* gene across diverse clinical and global isolates, supporting its essential role in *M.tb* survival and highlighting its potential as a target with a high barrier to resistance development. Structure-based virtual screening identified several GlgE binding candidates, among which VMP-7 emerged as the most promising lead compound through biological validation.

Experimental studies demonstrated that VMP-7 possesses significant anti-TB activity, with an MIC of 12.5 μg/mL and dose-dependent inhibition of intracellular *M.tb* growth within THP-1 derived macrophages. Importantly, VMP-7 retained activity against multidrug-resistant strains and exhibited minimal cytotoxicity at therapeutically relevant concentrations, indicating a favorable safety profile. Furthermore, combination treatment with standard anti-TB drugs enhanced bacterial clearance, suggesting the potential utility of VMP-7 as an adjunct therapeutic agent.

Collectively, these findings validate GlgE as a promising drug target and establish VMP-7 as a novel lead scaffold for anti-TB drug development. More broadly, this work demonstrates the value of integrating evolutionary conservation, structural biology, and experimental validation to identify drug targets and lead compounds with enhanced translational potential for TB therapy.

## Data Availability

The raw data supporting the conclusions of this article will be made available by the authors, without undue reservation.
