# Supplementary material for: Structure based discovery of VMP-7 as a promising GlgE targeting lead against Mycobacterium tuberculosis
Source: Front Microbiol. 2026 Sep 7;17:1909418. doi: 10.3389/fmicb.2026.1909418 (PMC13590571; doi:10.3389/fmicb.2026.1909418)
Supplement: Supplementary file 1 [file Table_1.DOCX]

**Supplementary table 1:** Chemical structures and molecular properties of the lead compounds (VMP-1 to VMP-12). Including Simplified Molecular Input Line Entry System (SMILES), Molecular formula and Molecular weight.

| **Sr. No** | **Name** | **2D Structure** | **SMILES** | **Molecular formula** | **Molecular weight** |
| --- | --- | --- | --- | --- | --- |
| 1 | **VMP-1** |  | O=C1[C@@]2(C([H])([H])C([H])([H])[H])C([H])([H])[N@@]3([H])C([H])([H])[C@]1(C([H])([H])C([H])([H])[H])C([H])([H])N(C2([H])[H])[C@@]3([H])[C@@]([H])(O[H])[C@@]([H])(O[H])[C@@]([H])(O[H])[C@@]([H])(O[H])C([H])([H])O[H] | C_17_H_31_N_2_O_6_ | 358.43 |
| 2 | **VMP-2** |  | O=C1[C@@]2(C([H])([H])[H])C([H])([H])[N@@]3([H])C([H])([H])[C@]1(C([H])([H])[H])C([H])([H])N(C2([H])[H])[C@@]3([H])[C@]([H])(O[H])[C@@]([H])(O[H])[C@@]([H])(O[H])C([H])([H])O[H] | C_14_H_2_N_2_O_5_ | 301.368 |
| 3 | **VMP-3** |  | [H]C([H])([H])C([H])(C([H])([H])[H])C([H])([H])[N@]1([H])C([H])([H])C([H])([H])OC([H])([H])[C@]2([H])C([H])([H])N([H])([H])C([H])([H])C([H])([H])[C@]12[H] | C_12_H_26_N_2_O | 214.354 |
| 4 | **VMP-4** |  | [H]N([H])([H])C([H])([H])[C@]1([H])C([H])([H])C([H])([H])C([H])([H])[N@]2([H])C([H])([H])C([H])([H])C([H])([H])C([H])([H])[C@]12[H] | C_10_H_2_N_2_ | 170.3 |
| 5 | **VMP-5** |  | O=C(c1c([H])c(OC([H])([H])[H])c([H])c([H])c1[H])C2=C([H])Oc3c2c(c(O[H])c([H])c3[H])C([H])([H])N4([H])C([H])([H])C([H])([H])C([H])([H])C([H])([H])C4([H])[H] | C_22_H_24_NO_4_ | 366.441 |
| 6 | **VMP-6** |  | O=C(N([H])[C@@]2([H])C([H])([H])C([H])([H])[N@@]([H])(C([H])([H])c1c([H])c([H])c([H])c([H])c1[H])C([H])([H])C2([H])[H])c5c([H])c3c(n([H])c4c([H])c([H])c([H])c([H])c34)c([H])n5[H] | C_24_H_26_N_4_O | 386.5 |
| 7 | **VMP-7** |  | O=C(N([H])[H])[C@@]4([H])C([H])([H])C([H])([H])N([H])(C([H])([H])[C@@]([H])(O[H])C([H])([H])Oc3c([H])c([H])c1c(OC(=O)C([H])=C1c2c([H])c([H])c([H])c([H])c2[H])c3[H])C([H])([H])C4([H])[H] | C_24_H_27_N_2_O_5_ | 423.494 |
| 8 | **VMP-8** |  | [H]N([H])([H])C([H])([H])C2(O[H])C([H])([H])C([H])([H])N([H])(C([H])([H])c1c([H])c([H])c([H])c([H])c1[H])C([H])([H])C2([H])[H] | C_13_H_22_N_2_O | 222.333 |
| 9 | **VMP-9** |  | [F]c1c([H])c([H])c([H])c(c1[H])C([H])([H])[N@@]4([H])C([H])([H])C([H])([H])[C@]([H])(C([H])([H])N([H])C(=O)C([H])([H])C2=C3[C@]([H])(N(C2=O)C([H])([H])[H])C([H])([H])C([H])([H])C([H])([H])C3([H])[H])C([H])([H])C4([H])[H] | C_24_H_33_FN_3_O_2_ | 414.547 |
| 10 | **VMP-10** |  | [Cl]c4c([H])c1c(n([H])c2c1nc([H])n(c2=O)C([H])([H])C([H])([H])C([H])([H])n3c([H])c([H])n([H])c3[H])c([H])c4[H] | C_16_H_15_ClN_5_O | 328.784 |
| 11 | **VMP-11** |  | O=c1n(c(=O)c(c(O)n1C([H])([H])[H])[S](C([H])([H])[H])C([H])([H])C([H])([H])[C@@]([H])(C(O)=O)N([H])([H])[H])C([H])([H])[H] | C_11_H_19_N_3_O_5_S | 305.36 |
| 12 | **VMP-12** |  | [H]C([H])([H])Oc1c([H])c([H])c2c5c1O[C@]4([H])[C@@]([H])(O[H])C([H])=C([H])[C@@]3([H])[C@]([H])(C2([H])[H])N(C([H])([H])C([H])([H])[C@@]345)(C([H])([H])[H])C([H])([H])[H] | C_19_H_24_NO_3_ | 314.408 |
